# The Mechanism Exploration of Traditional Chinese Medicine's “Different Treatments for Same Disease” Concept in Osteoporosis Therapy: A Serum Metabolomics Study

**DOI:** 10.1111/jcmm.70662

**Published:** 2025-07-02

**Authors:** Jingyuan Wen, Xuefeng Li, Zhen Wu, Liu Jiangyuan, Guanyin Wang, Xu Wang, Zhengsheng Bao, Yang Yu, Pinger Wang, Zhenyu Shi, Bing Xu, Yunhuo Cai, Hongting Jin, Jiali Chen

**Affiliations:** ^1^ Institute of Orthopaedics and Traumatology of Zhejiang Province The First Affiliated Hospital of Zhejiang Chinese Medical University (Zhejiang Provincial Hospital of Chinese Medicine) Hangzhou China; ^2^ Department of Orthopedic Surgery Tongde Hospital of Zhejiang Province Hangzhou China; ^3^ Department of Orthopedics the Second Affiliated Hospital of Zhejiang Chinese Medical University, Zhejiang Chinese Medical University Hangzhou China; ^4^ The Affiliated Wenzhou Hospital of Integrated Chinese and Western Medicine The First Affiliated Hospital of Zhejiang Chinese Medical University Hangzhou China; ^5^ Department of Rehabilitation The Third Affiliated Hospital of Zhejiang Chinese Medical University Hangzhou China

**Keywords:** different treatments for same disease, invigorating blood, osteoporosis, strengthening spleen, tonifying kidneys, traditional Chinese medicine

## Abstract

The “different treatments for same disease” is an important concept of traditional Chinese medicine (TCM) therapy. In TCM, osteoporosis (OP) treatment is aimed at invigorating blood, strengthening spleen, and tonifying kidneys, and their typical herbs are *Angelica sinensis* (*Oliv.*) *Diels* (Danggui, DG), *Poria cocos* (*Schw.*) *Wolf* (Fuling, FL), and *Achyranthes bidentata Blume* (Niuxi, NX). Nevertheless, molecular mechanisms of these different therapies of OP under the concept of “different treatments for same disease” are still unclear. The objective of this study was to identify the related metabolites and biological processes in these three distinct therapeutic approaches for osteoporosis, by using serum metabolomics analysis. A model of postmenopausal OP (PMOP) was created using bilateral ovariectomized rats and then administered with DG, FL, or NX for 12 weeks. To assess the efficacy of the three treatments, we performed gross pathology evaluation, micro‐computed tomography (micro‐CT) scan, bone‐strength test, and histopathologic examination. The results demonstrated that the treatment groups improved weight, rectal temperature, and 24‐h urine output when compared to the model group. Furthermore, the PMOP models exhibited significant increases in bone strength, bone mass, and physical bone parameters after three distinct treatments. Serum metabolomics analysis subsequently showed that DG was predominantly associated with glycerophospholipids, prenol lipids, and steroid lipid metabolism. FL was primarily linked to glycerophospholipid and amino acid metabolism. The primary metabolisms associated with NX include sphingolipid, glycerophospholipid, amino acid, and purine metabolisms. In conclusion, the DG, FL, and NX herbs effectively alleviate PMOP by regulating lipid metabolism, while FL is also involved in amino acid metabolism and NX in amino acid and purine metabolisms. Our results provide biological evidence for the TCM principle of “different treatments for same disease”.

## 1 Introduction

Postmenopausal osteoporosis (PMOP), caused by oestrogen withdrawal in women following menopause, is the most prevalent osteoporosis. It is a systemic bone metabolic disease, resulting from an imbalance between osteogenic and osteoblastic homeostasis. PMOP is characterised by decreased bone mineral density, destruction of bone microstructure, and an increased risk of fragility fractures [1]. It is estimated that approximately half of all postmenopausal women worldwide are affected by osteoporosis, with a prevalence of fractures in patients with osteoporosis as high as 40% [2, 3]. Since the current clinical medicines lack safety and efficacy in treating PMOP, there is an urgent need to develop novel treatment strategies for PMOP.

Traditional Chinese medicine (TCM) is well known for its long history, good efficacy, and low side effects [4]. As an important part of the theory of TCM, “different treatments for same disease” has guiding significance for the treatment of various diseases [5, 6, 7]. Under the guidance of this concept, the primary objective of osteoporosis treatment is to invigorate blood, strengthen spleen, and tonify kidney [3, 8, 9, 10]. In the TCM theory, the kidney is a viscera which stores vital essence and is responsible for the activities of bones [11, 12]. A TCM classic states that in older people and post‐menopausal women, the kidney is insufficient [13, 14], so strengthening kidney alleviate related symptoms, including osteoporosis [15, 16]. Additionally, in TCM, the spleen controls transportation and transformation, so spleen deficiency can impact blood circulation and absorption of nutrients, resulting in blood stasis, which in turn affect bone health [17]. Previous studies conducted with ovariectomized (OVX) animals have also demonstrated that tonifying kidney, strengthening spleen, and invigorating blood drugs have the effect of increasing bone density [15, 18, 19, 20].

According to ancient books, the Pharmacopoeia of the People's Republic of China, and relevant modern research literatures [18], *Angelica sinensis* (*Oliv*.) *Diels* (Danggui, DG), *Poria cocos* (*Schw*.) *Wolf* (Fuling, FL), and *Achyranthes bidentata Blume* (Niuxi, NX) were selected as representative herbals for invigorating blood, strengthening spleen, and tonifying kidney treatments, respectively. It is recorded in the classic Chinese medicine books that DG has the effect of nourishing and invigorating blood and has been used for invigorating blood circulation in China for more than 2000 years [21]. Meanwhile, modern medical researches have also confirmed the remarkable effect of DG on blood circulation and treatment of anaemia as well as osteoporosis [22, 23, 24]. In TCM, the spleen prefers dryness to dampness [25]. FL is a dampness‐clearing drug in TCM, which works by removing abnormal accumulation of liquid water in the body and body cavities, so FL can reduce the burden on the spleen and restore its normal function [26, 27]. The kidney tonifying effects of NX are widely recognised in TCM clinical practice [28]. Modern pharmacological studies have also shown that it possesses kidney‐protective and anti‐osteoporosis activities [29, 30, 31]. Although the feasibility of these ifferent treatments for osteoporosis has been confirmed [10], the underlying mechanisms remain elusive.

Herbal medicine treats diseases effectively, with multiple components, multiple targets, and multiple pathways as its typical features [32]. At present, the main challenge of TCM development is how to clarify the theories of TCM from the perspective of modern science. Metabolomics, like genomics and proteomics, is a new subject that has arisen in recent years. Metabolomics, as on the basis of the mass spectrometry and advanced analytical approaches, provide high‐throughput qualitative and quantitative information of metabolites in the body, with the objective of revealing pathological and physiological states at the holistic level [33]. Thus, metabolomics analysis is an important tool in the field of TCM research. Wang et al. [34, 35] elucidated the mechanism of action of herbs with different properties on hyperthyroidism and hypothyroidism using metabolomics and network pharmacology target prediction. Li et al. [36] demonstrated that puerarin treats osteoporosis by modulating the metabolism of phospholipids and the biosynthesis of unsaturated fatty acids through a combination of animal experiments and serum metabolomics analysis. The above studies suggest that metabolomics analysis is highly compatible with TCM research and is an important tool for us to explore the “different treatments for same disease” theory of TCM in PMOP treatment.

The PMOP model rats were treated with DG, FL, and NX. The results of gross pathology evaluation, micro‐computed tomography (micro‐CT) scan, biomechanical test, and histopathologic examination showed that DG, FL, and NX were effective in the treatment of osteoporosis. Furthermore, metabolomics analysis showed that DG was mainly related to lipid metabolism and FL was primarily related to lipid and amino acid metabolisms. NX is related to lipid, amino acid, and purine metabolisms. Altogether, under “different treatments for same disease” theory, invigorating blood, strengthening spleen and tonifying kidney possess the common mechanisms, while specific biological functions may also exist between them.

## 2 Materials and Methods

### 2.1 Animals

Forty 10‐week‐old female Sprague–Dawley rats (weighing approximately 230–250 g) were purchased from the Experimental Animal Center of Zhejiang University of Traditional Chinese Medicine (Hangzhou, China). All rats were placed in a 12 h/12 h light/dark cycle at a temperature of 20°C ± 3°C and a relative humidity of 60% ± 10%. Animals had free access to food and sterile water. The experimental procedures were conducted in accordance with the Guide for the Care and Use of Laboratory Animals (NIH Publication No. 80–23, revised 1978) and approved by the Animal Care and Use Committee of Zhejiang University of Traditional Chinese Medicine (LZ12H27001). After 1 week of acclimatisation, the animals were randomly divided into the sham operation group, model group, DG group, FL group, and NX group. Except for the sham operation group, the other four groups underwent bilateral ovariectomy.

### 2.2 The Construction of OVX Rats

**FIGURE 1:**
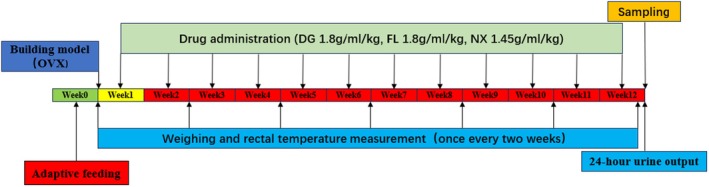
OVX rat modelling and drug intervention flow chart.

All in all, SD rats were anaesthetised by intraperitoneal injection of zoletyl (40 mg/kg). The rats were placed in a prone position. The surgical area was disinfected before the operation. An incision of about 1 cm was made 1.5 cm above the ilium and 2 cm next to the spine. The fat tissue was gently grasped with curved tweezers and slowly pulled out to reveal the ovaries and fallopian tubes. Next, after ligation of the fallopian tubes, the ovaries were removed. The fat tissue was then pushed back into the abdominal cavity, and skin tissue was sutured. Penicillin was used for 3 days after the operation to prevent infection. Drug administration was started on the second postoperative day. The DG, FL, and NX groups were given the corresponding drugs by gavage, 5 mL/day. The same volume of saline was given to the sham operation and model groups. Gavage was continued for 12 weeks (Figure 1).

### 2.3 Preparation of Chinese Medicine Liquid

Soak the herbs in 10 times the volume of pure water for 1 h, then heat them to boiling point over high heat, then continue heating over low heat for 1 h, and filter out the dregs. Add 10 times the volume of purified water to the dregs and decoct again according to the above method. The filtrate obtained from the two decoctions was thoroughly mixed, and then the filtrate was evaporated and concentrated under reduced pressure. The concentrated liquid was then stored at −20°C for future use. All the herbs were purchased from the preparation laboratory of Zhejiang University of TCM. Detailed information of the herbs is shown in Table 1.

**TABLE 1:** Detailed information of the herbs.

Chinese herb	Full taxonomy name	Abbreviations	Drug concentration (g/mL)	Gavage dose (mL)	Parts used
DangGui	*Angelica sinensis* (Oliv.) Diels	DG	0.16	2	Root
FuLing	Poria cocos (Schw.) Wolf	FL	0.2	2	Dried mycorrhizae
NiuXi	*Achyranthes bidentata* Bl	NX	0.16	2	Root

### 2.4 Chemicals, Reagents, and Instruments

Methanol (A‐456‐4, USA), acetonitrile (955‐4, USA), and formic acid (A17‐50, USA) were purchased from Fischer Corporation (USA). DG, FL, and NX were kindly provided by Peking Tongrentang Company Limited (Beijing, China). All voucher specimens were kept at Zhejiang University of Traditional Chinese Medicine. The reagents used in the experiments included Alcian Blue 8G (A5268‐25G), Orange G (1963‐15‐8), haematoxylin (G1100), and eosin (G1100). Experimental apparatus included Micro‐Computed Tomography (Skyscan 1176, Bruker Micro‐CT N.V, Kontich, Belgium), Automatic Dewatering Machine (Leica TP1020, Leica Biosystems, Buffalo Grove, USA), UltiMate 3000 UPLC system (Thermo Fisher Scientific, Bremen, Germany), and Triple TOF 6600 (SCIEX, Framingham, MA, USA).

### 2.5 Collection of Weight and Rectal Temperature Data

The weights and rectal temperatures of the rats were measured and recorded at fixed time points every 2 weeks, and the changes in the weights and rectal temperatures of the rats in each group were observed. The 24‐h urination of each rat was collected separately through the metabolic cage 2 days before sampling. The detailed process is shown in Figure 1.

### 2.6 Collection and Preparation of Samples

The rats were anaesthetised by inhalation of isopentane gas, and when the rats were unconscious, approximately 10 mL of blood was withdrawn from the heart with a syringe and allowed to stand for 1 h at room temperature, centrifuged at 4500 rpm for 10 min, and the supernatant was collected and stored at −80°C for subsequent analysis. The rats were euthanised, and the right and left femurs were removed and immersed in 4% paraformaldehyde for 5 days for fixation. Histology and micro‐CT were then performed. The right and left tibiae were removed for biomechanical testing. In addition, liver and kidney tissues were fixed in 4% paraformaldehyde for 4–8 h and then dehydrated and embedded for further experiments.

### 2.7 BMD and Micro‐CT Analysis

Structural changes in bone volume and trabeculae of the distal femur were analysed using micro‐computed tomography to determine their skeletal characteristics. Bone mineral density (BMD, g/cm^3^) was obtained by contour volume of interest (VOI) images, and skeletal morphological characteristics included bone volume to total volume ratio (BV/TV, %), trabecular number (Tb.N, 1/mm), trabecular structural modelling index (SMI), and trabecular separation (Tb.Sp, μm).

### 2.8 Haematoxylin–Eosin Staining

Morphological and structural changes of liver and kidney tissues were detected by haematoxylin–eosin (H&E) staining. Liver and kidney tissue samples were collected and fixed with 4% paraformaldehyde for 6–8 h. After dehydration (Leica TP1020, Leica Biosystems, Buffalo Grove, USA), the tissues were embedded in paraffin. Tissue sections of 3 μm thickness were cut from the paraffin blocks using a standard slicer (Leica biosystems, Buffalo Grove, USA), then flattened and heat‐fixed on cationic slides and stained for H&E. In short, the sections were deparaffined with xylene I, xylene II, and xylene III for 10 min each, then soaked in 100%, 100%, 95%, 85%, and 75% alcohol for 5 min in turn, and finally rinsed with pure water three times. The section was stained with haematoxylin for 3–5 min to make the nucleus blue. The slices were treated with 1% hydrochloric acid alcohol for 3–5 s and then rinsed with pure water. The slices were incubated in 0.5% ammonia solution for 20 s. After rinsing, the sections were placed in eosin staining solution for 1–2 min to stain the cytoplasm red. The stained sections were dehydrated in 75%, 85%, 95%, 100% and 100% alcohol for 3 min. Then they were transparent with xylene I, xylene II, and xylene III for 5 min each. H&E staining is completed by dropping neutral gum onto the slice, covering the cover glass to avoid bubbles and allowing it to dry naturally. Five sections per sample were selected for observation under a 400× light microscope and photographed.

### 2.9 Alisin Blue Haematoxylin/Orange G (ABH) and Tartrate‐Resistant Acid Phosphatase (TRAP) Staining

Femoral samples were fixed in 4% paraformaldehyde for 5 days, decalcified by immersion in 14% EDTA solution for 3 months, and then dehydrated and paraffin‐embedded. The samples were cut into 3 μm thick tissue sections for Alisin Blue Haematoxylin/Orange G (ABH) staining using a standard microtome. Briefly, sections were deparaffinised, washed three times in PBS, immersed in 1% hydrochloric acid alcohol for 30 s, and then immersed in Alisin Blue dye for 1 h. The samples were immersed in 1% hydrochloric alcohol for 5 s, washed in purified water, immersed in 0.5% ammonia for 15 s, washed again, immersed in 95% alcohol for 1 min, immersed in Orange G dye for 90 s, washed, and dried at 37°C. The samples were then removed from the microscope and observed under a microscope. Finally, the changes in bone microstructure were observed under a microscope and photographed.

Prepare the TRAP staining working solution according to the instructions of the kit. Drop the prepared staining solution onto the sample to ensure that the sample is completely covered. Then place the slide in a wet box and incubate it at 37°C for 60 min. After the staining is completed, gently rinse the sample with distilled water to remove the excess staining solution. Subsequently, immerse the sample in the methyl green counterstaining solution for counterstaining for 3 min. After the counterstaining is finished, rinse the sample again with distilled water to remove the excess counterstaining solution. Dehydrate and clarify the sections, seal them with neutral resin, let them air dry, and then observe them under a microscope.

### 2.10 Immunohistochemistry Staining

Alkaline phosphatase (ALP, Arigo, ARG57422, 1:200) in rat femur samples was detected by immunohistochemistry. Runt‐associated transcription factor 2 (Runx2, Abcam, ab236639, 1:200), fatty acid–binding protein 4 (Fabp4, Abcam, ab92501, 1:200), and leukocyte differentiation antigen 36 (Cd36, Huabio, ET1701‐24, 1:200) expression levels were assessed. Sections were deparaffinised in xylene (3 × 10 min) and hydrated through a graded ethanol series (100%, 100%, 95%, 85%, 75%; 5 min each), followed by distilled water rinsing. Antigen retrieval was achieved by immersing the sections in 10 mM sodium citrate buffer (pH 6.0) at 65°C for 4 h. Endogenous peroxidase activity was blocked with 3% H_2_O_2_ (25°C, 10 min), followed by three 3‐min PBS washes. Non‐specific binding was inhibited with 10% normal goat serum (37°C, 30 min). After being washed three times with PBS, primary antibodies were applied and incubated at 4°C overnight. Species‐matched secondary antibodies were subsequently added and incubated at 37°C for 30 min. Colour development was performed using DAB chromogen (Dako) under microscopic monitoring. Nuclear counterstaining was carried out with haematoxylin for enhanced morphological visualisation. Finally, sections were dehydrated through an ascending ethanol gradient (75%–100%), cleared in xylene (3 × 5 min), and mounted with neutral gum under cover slips. Observe and record the results under a microscope.

### 2.11 UPLC‐Q‐TOF/MS Analysis

The collected samples were thawed on ice, and metabolites were extracted with 50% methanol buffer. Briefly, 20 μL of sample was extracted with 120 μL of pre‐cooled 50% methanol, vortexed for 1 min, and incubated at room temperature for 10 min; the extracts were stored overnight at −20°C and centrifuged at 4000 g for 20 min before transferring the supernatant to a new 96‐well plate. Samples were stored at −80°C prior to LC–MS analysis, and 10 μL of each extract was taken to prepare a mixed QC sample. Finally, 2 μL of the supernatant from each sample was injected into the UPLC‐Q‐TOF/MS system for metabolomics analysis.

All samples were collected by the LC–MS system according to the instrument instructions. First, all chromatographic separations were performed on an UltiMate 3000 UPLC system. The reversed‐phase separation was performed on an ACQUITY UPLC T3 column (100 × 2.1 mm, 1.8 μm, Waters, Milford, USA). The column chamber was maintained at 40°C with the addition of 5 mM ammonium acetate and 5 mM acetic acid and solvent B (acetonitrile). The low flow rate was 0.3 mL/min, and the mobile phase was solvent A. The gradient elution conditions were set as follows: 0~0.8 min, 2% B; 0.8~2.8 min, 2%~70% B; 2.8~5.6 min, 70%~90% B; 5.6~6.4 min, 90%~100% B; 6.4~8.0 min, 100% B; 8.0~8.1 min, 100%~2% B; 8.1~10 min, 2%B.

Metabolites eluting from the column were detected using a high‐resolution tandem mass spectrometer, Triple TOF 6600, with the Q‐TOF operating in positive and negative ion modes. The curtain gas was set to 30 PSI, ion source gas 1 was set to 60 PSI, ion source gas 2 was set to 60 PSI, and the interface heater was set to 500°C. Q‐TOF was used to detect metabolites eluting from the column. For positive ion mode, the ion spray float voltage was set to 5000 V. For negative ion mode, the ion spray float voltage was set to −4500 V. Mass spectrometry data were acquired in IDA mode with TOF masses ranging from 60 to 1200 Da. Survey scans were acquired within 150 ms and up to 12 scans were collected if the threshold of 100 counts per second (counts/s) was exceeded with a 1+ charge. Up to 12 product ion scans were collected if the threshold of 100 counts per second (counts/s) was exceeded and the 1+ charge state was present. Dynamic exclusion was set to 4 s. Mass accuracy was calibrated every 20 samples during acquisition.

### 2.12 Data Processing and Metabolic Profiling

Peak picking, peak grouping, retention time correction, secondary peak grouping, and isotope and adduct labeling were performed on the acquired mass spectrometry data using XCMS software. LC–MS raw data files were converted to mzXML format and then processed using the XCMS, CAMERA, and metaX toolboxes implemented in the R software. Ions were identified by combining retention time (RT) and m/z data. The intensity of each peak was recorded, and a three‐dimensional matrix was generated containing any specified peak index (retention time‐m/z pair), sample name (observation) and ion intensity information (variable). The metabolites were annotated using the online KEGG, HMDB database, and the exact molecular mass data (m/z) of the samples were matched to the data in the database. If the mass difference between the observed and database values is less than 10 ppm, the metabolite will be annotated, and the molecular formula of the metabolite will be further identified and verified by isotopic distribution measurements. We also used the in‐house database to validate metabolite identifications. Statistical analysis of the data was mainly done by R software (version 4.0); the raw intensity values of the proteins were median normalised, cluster heatmaps were drawn by the R package pheatmap, PCA analysis, and analysis of significantly different proteins were done by the R package metaX, PLS‐DA analysis was done by the R package ropls, and the VIP values of each variable were calculated, and correlations were done by the Pearson's Poll of R package cor. The correlation analysis was performed by the Pearson correlation coefficient of R package cor, and the final significantly different metabolites were screened out by the *t*‐test with the three conditions of *p*‐value < 0.05, the multiplicity of difference > 1.2, and the VIP calculated by PLSDA analysis satisfied at the same time. Differential enrichment analysis of the KEGG pathway was performed on the basis of the hypergeometric test, and the functional entries with *p*‐value < 0.05 from the statistical test were the functional entries significantly enriched for differential proteins.

### 2.13 Statistical Analysis

All data were analysed with SPSS 26.0 software (IBM; New York, NY, USA). Statistical significance was assessed by one‐way analysis of variance (ANOVA) and compared with the control of each experiment (*p* < 0.05).

## 3 Results

### 3.1 Therapeutic Effects of DG, FL, and NX on Weight, Rectal Temperature, and 24‐h Urination Volume in Model Rats

**FIGURE 2:**
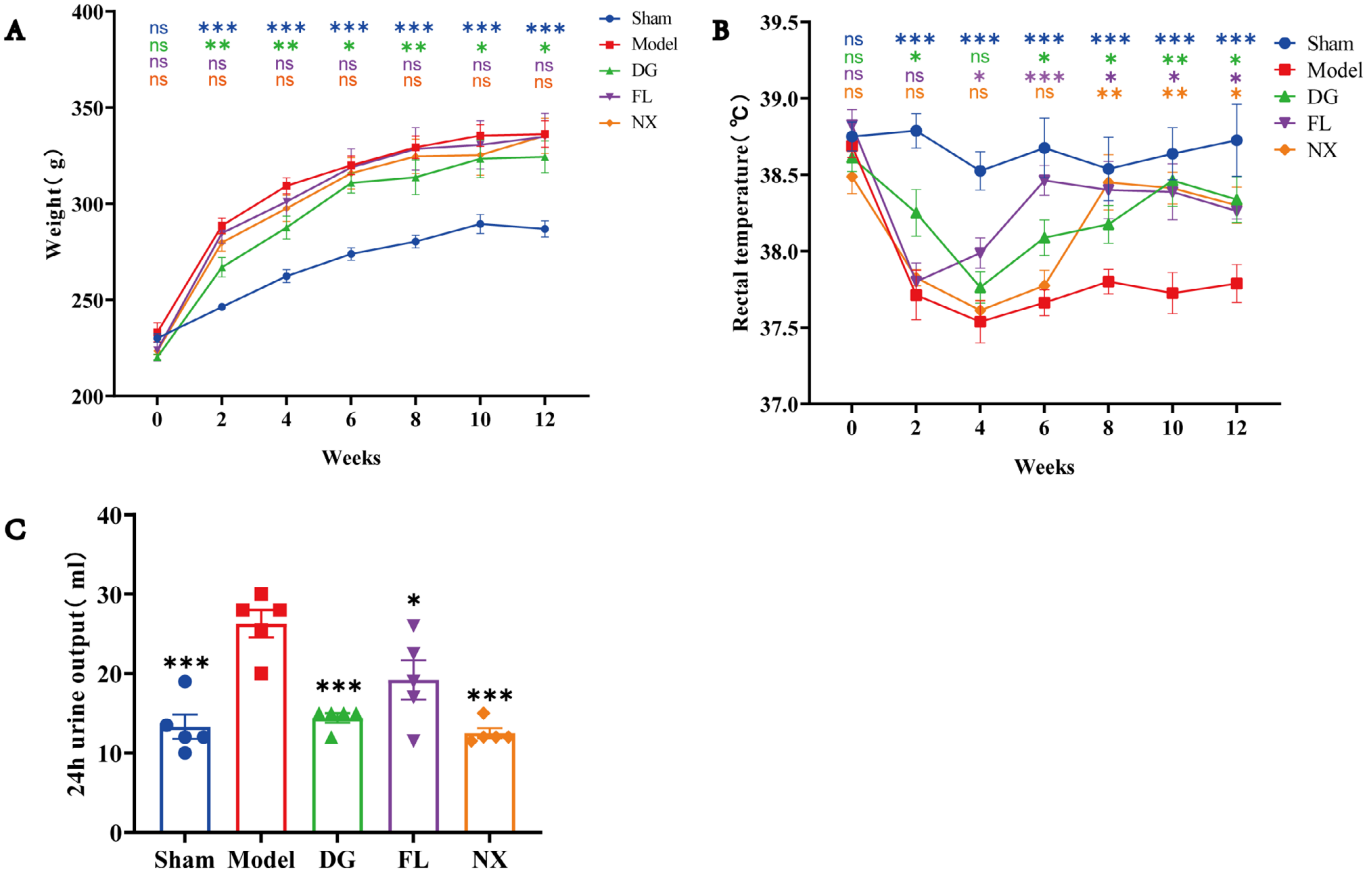
Trends of body weight (A), rectal temperature (B), and 24‐h urine output (C) of rats in each group. *p < 0.05, **p < 0.01 and ***p＜0.001 for comparison with model group.

Weight, rectal temperature, and 24‐h urine volume of rats in each group were analysed, and the results are shown in Figure 2. We found that compared with the sham group, the weight of rats in the model group was significantly increased. After DG treatment, the speed of weight gain in model rats slowed down (Figure 2A). However, there were no significant changes in weight in the FL and NX groups when compared with the model group (Figure 2A). Compared with the sham group, rectal temperature of rats in the model group decreased continuously during the first 2 weeks after OVX modelling, but became stable during the following weeks (Figure 2B). After 8 weeks of administration, rectal temperature in the NX group was significantly higher than that in model rats, and after 6 weeks of administration, rectal temperature in the FL group and DG group was significantly higher than that in model rats (Figure 2B). The 24‐h urination of rats was quantified, and the results showed that compared with the sham group, the 24‐h urination volume of rats in the model group was significantly increased, while DG, FL, and NX treatments inhibited this abnormal change (Figure 2C).

In addition, histopathological changes of liver and kidney tissues were observed (Figure S1). Compared with the sham group, except for the slight increase of hepatocyte adipocytes in the model group, the morphology and tissue structure of liver and kidney tissue in the other groups were normal, and no pathological changes were observed (Figure S1A,B).

### 3.2 Changes in Bone Microstructure and Biomechanical Parameters in OVX Rats Following Three Distinct Herbal Therapies

**FIGURE 3:**
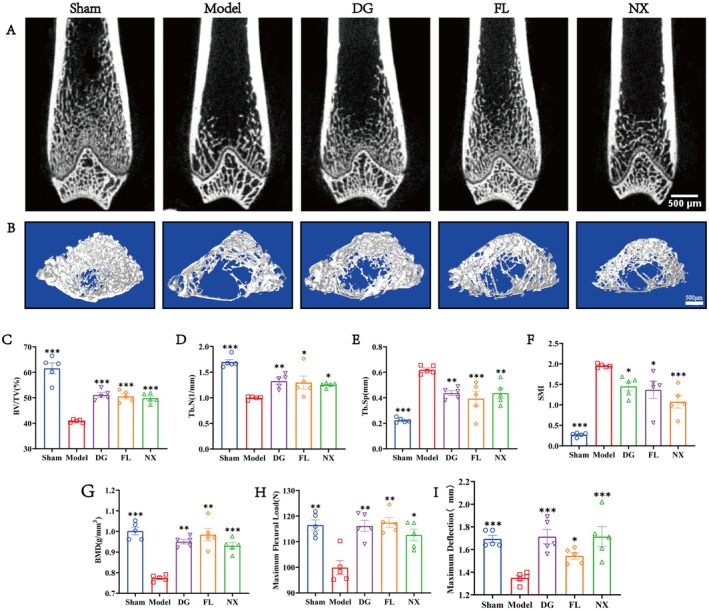
Changes in the bone structure in OVX rats. (A, B) Representative micro‐CT images. Quantification of microstructural parameters including BV/TV (C), Tb.Sp (D), BMD (E), Tb.N (F), and SMI (G). Biomechanical parameters including maximum deformation (H) and maximum bending load (I). *p < 0.05, **p < 0.01 and ***p < 0.001, ns indicates that the difference is not statistically significant. Scale bar = 100 μm.

The distal femur of OVX rats was analysed for bone microstructure using micro‐CT. The 2D and 3D structural drawings demonstrated that the amount of bone in the medullary cavity was significantly reduced following modelling (Figure 3A,B). Specifically, compared with the sham group, BMD, BV/TV, and Tb.N of the distal femur of rats in the model group were significantly decreased, while Tb.Sp was significantly increased (Figure 3C–F). Compared with the model group, BMD, BV/TV, and Tb.N of the distal femur in the DG, FL, and NX groups were significantly increased, while Tb.Sp was significantly decreased (Figure 3C–F). In addition, the trabecular microstructure was also analysed, and the results showed that SMI in the model group was significantly higher than that in the sham group. After three distinct herbs administrations, SMI was significantly reduced (Figure 3G).

Additionally, a three‐point bending test was conducted to assess the pertinent biomechanical parameters of the bone. The results demonstrated that the maximum bending load and maximum flexion displacement of the tibia were significantly lower in the model group compared to the sham group (Figure 3H,I). Following the administrations of DG, FL, and NX, the maximum bending load and maximum flexion displacement were significantly elevated (Figure 3H,I). In summary, the three drugs delay the bone loss and improve the mechanical properties of bone in model rats.

### 3.3 Three Distinct Herb Treatments Delayed Bone Loss and Fat Accumulation in OVX Rats

**FIGURE 4:**
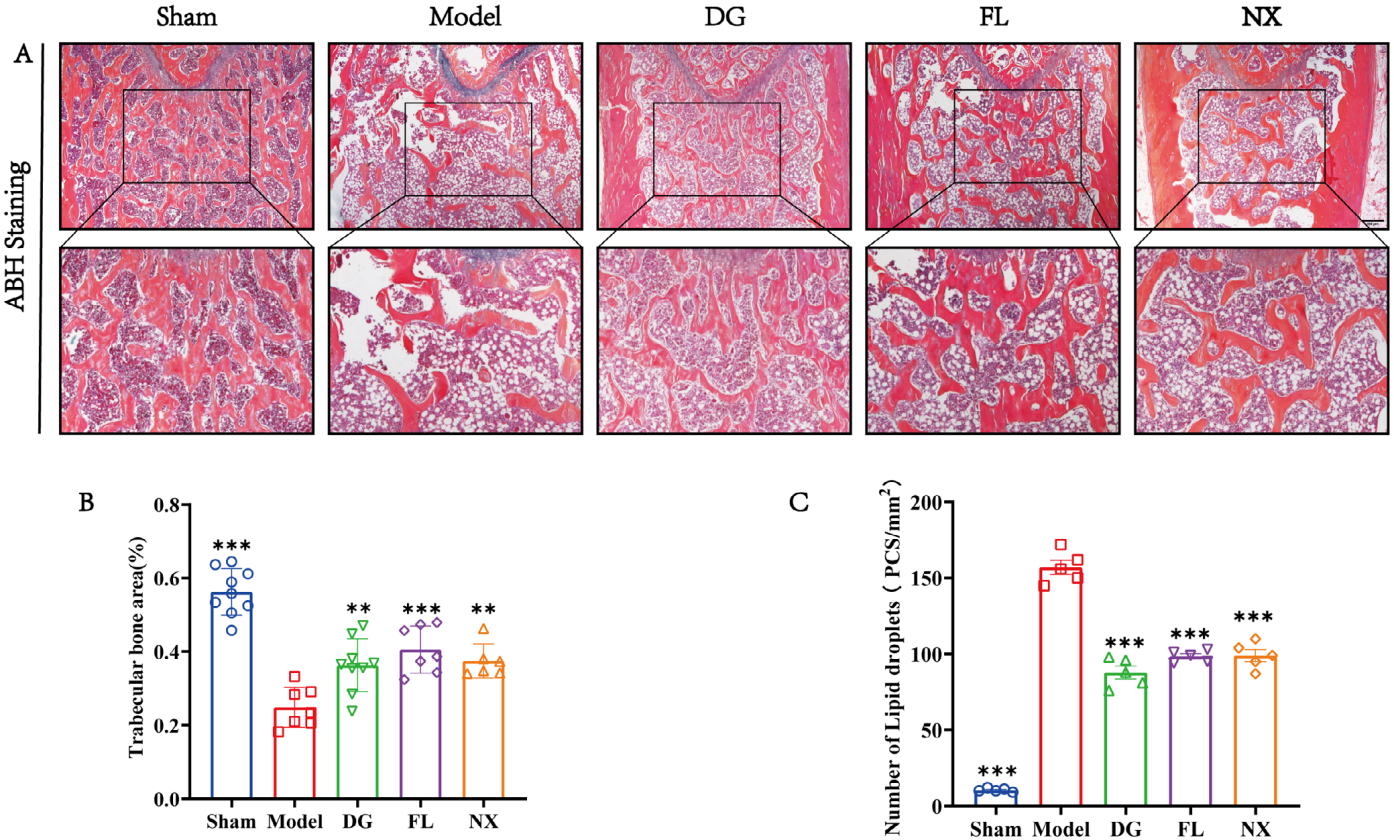
Three herbs treatments prevented bone loss in OVX rats. (A) ABH staining of distal femur. (B) Bone trabecular area (%). (C) Number of lipid droplets. Black **p < 0.01 and ***p < 0.001, ns indicates not significant difference. Scale bar = 50 μm.

To verify the efficacy of the three herbs against PMOP, we examined the femur samples of OVX rats histologically by ABH staining. Compared with the sham operation group, the trabecular bone density in the distal femoral medullary cavity of the model group was significantly reduced, the trabecular structure was disorganised (Figure 4A,B), and a large number of fat vacuoles were formed in the trabecular space (Figure 4A–C). These data suggest a severe imbalance between osteogenesis and adipogenesis in the model group of rats. Compared with the model group, the DG, FL, and NX groups showed a significant increase in bone trabecular area and a significant decrease in the number of fat vacuoles in the medullary cavity (Figure 4A–C).

**FIGURE 5:**
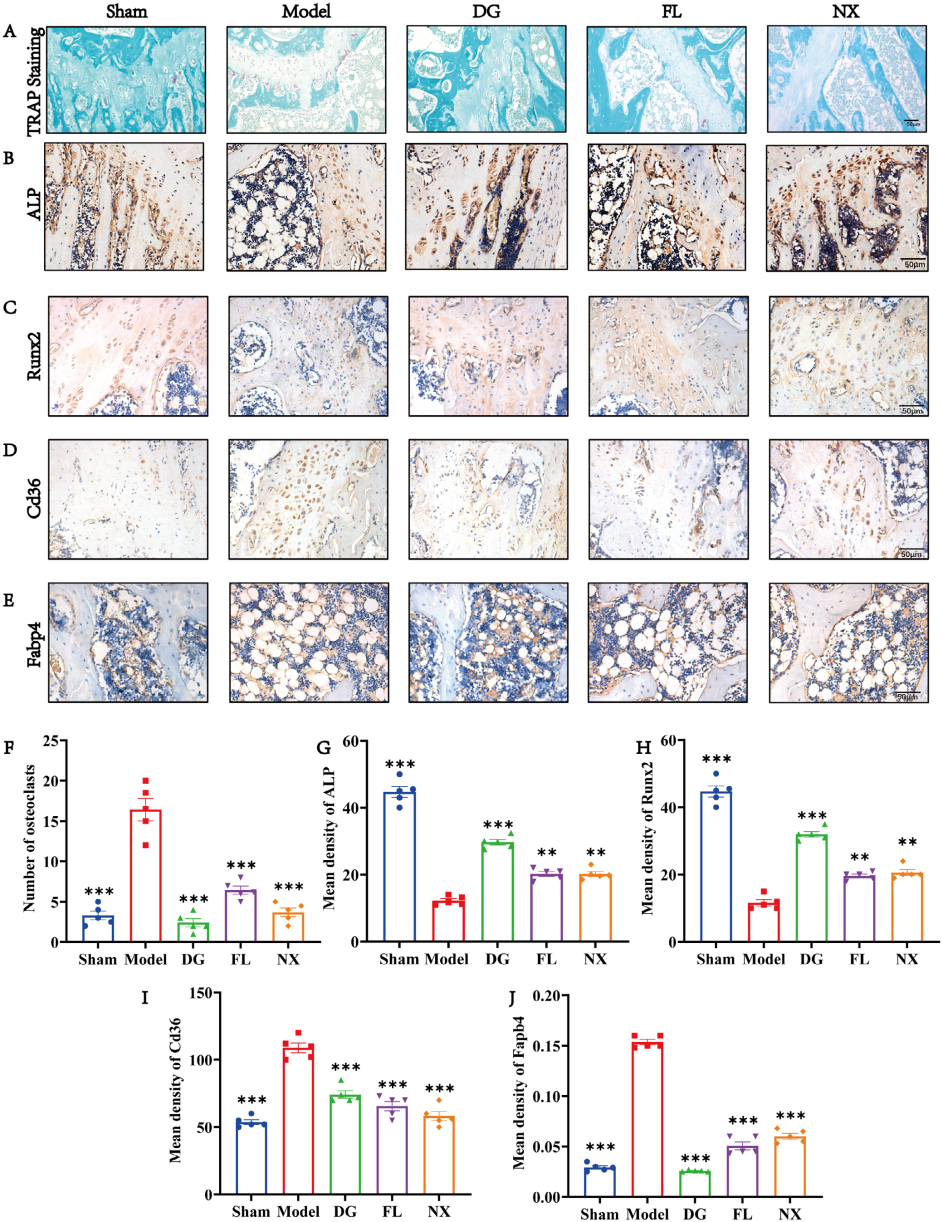
Expression levels of osteogenesis, osteoclastogensis and lipogenesis related proteins in OVX rats after DG, FL, and NX treatment. (A–E) Representative TRAP staining and immunohistochemical images as well as quantification of TRAP, ALP, Runx2, Fabp4, and Cd36. *p < 0.05, **p < 0.01, ***p < 0.001. Scale bar = 50 μm.

To further verify the effects of the three drugs on osteogenesis, osteoclastogenesis, and fat formation, we performed immunohistochemistry and TRAP staining. The results showed that compared with the sham operation group, the number of osteoclasts in the model group was significantly increased, and after DG, FL, and NX treatment, the osteoclasts waswere significantly decreased (Figure 5A,F). Immunohistochemical results showed that compared with the sham group, the expression levels of bone indexes ALP and Runx2 in the model were significantly decreased, but their expressions were significantly increased after treatment with DG, FL, and NX (Figure 5B,C,G,H). In terms of lipid formation, Fabp4 and Cd36 in the model group were significantly higher than those in the sham group. However, DG, FL, and NX treatments down‐regulated the expression levels of Fabp4 and Cd36 (Figure 5D,E,I,J). Thus, the DG, FL, and NX may suppress OVX‐induced imbalance between adipogenesis, osteoclastogenesis, and osteogenesis.

### 3.4 Analysis of Serum Differential Metabolites in DG‐Treated OVX Rats

**FIGURE 6:**
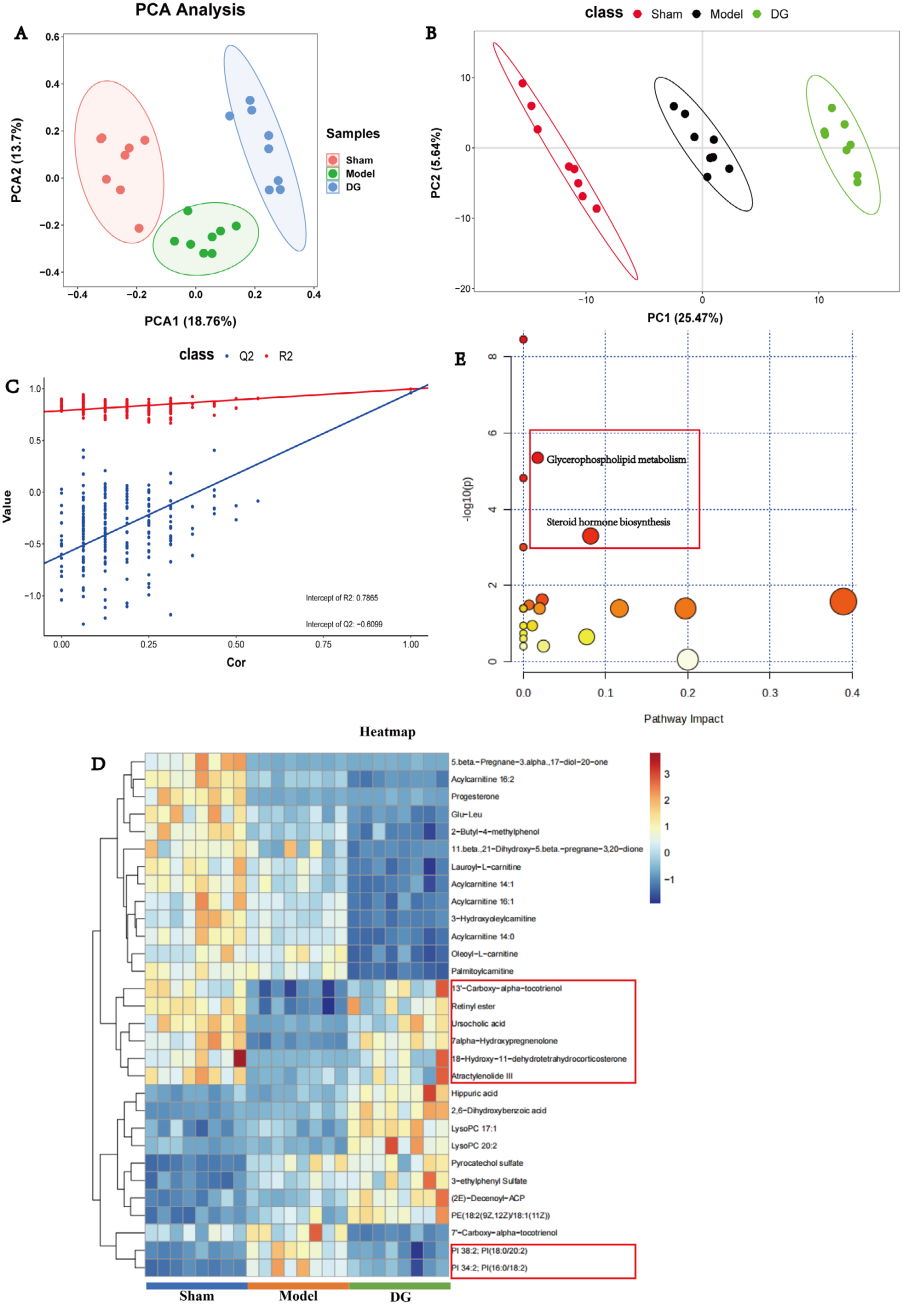
Metabolomic analysis of DG treatment, model, and sham groups. (A) PCA analysis of DG; (B, C) PLS‐DA of DG; (D) heat map and cluster analysis of differential metabolites. (E) Functional enrichment analysis of differential metabolites.

PCA clearly showed differences in metabolic profiles between the DG, model and sham rat groups (Figure 6A), indicating that DG treatment induces systemic metabolic changes in OVX rats. To determine the accuracy of this result, PLS‐DA, a supervised discriminative method, was conducted, which can filter out non‐essential variables and significantly improve the accuracy of classification. The results of PLS‐DA showed the significant within‐group clustering among the DG, sham and model groups (Figure 6B,C). In addition, all differential metabolites between these three groups, identified by PLS‐DA analysis in accordance with a predefined criterion (VIP > 1; *p* < 0.05), are shown in Table 2. Furthermore, the top 30 differential metabolites were selected for cluster heat map analysis (Figure 6D). Among them, we found 6 differential metabolites that were decreased following modelling while creased after DG treatment, namely, 13′‐carboxyl‐alpha‐tocotrienol, retinol ester, urcholic acid, 7‐alpha‐hydroxypregnenolone, 18‐hydroxy‐11‐dehydrotetrahydrocorticosterone, and aletholactone III. Besides, two differential metabolites that were up‐regulated following modelling while down‐regulated after DG treatment, namely, PI 38:2; PI (18:0/20:2) and PI 34:2; PI (16:0/18:2). We found that these metabolites were mainly related to glycerolipid metabolism, prenol lipid metabolism, and steroid metabolism.

**TABLE 2:** All the differential metabolites screened among Sham, Model, and DG groups (VIP > 1; *p* < 0.05).

Name	MZ	RT	Formula	Superclass	Class	KEGG	*p*	VIP
(1R)‐Chrysanthemolactone	186.16	3.62	C10H16O2	Organoheterocyclic compounds	Lactones	NA	0.03	1.17
(2E)‐Decenoyl‐ACP	128.07	1.28	C6H11NO2	Organic acids and derivatives	Carboxylic acids and derivatives	C03969	0.00	1.24
(3beta, 8beta)‐3‐Hydroxy‐7(11)‐eremophilen‐12,8‐olide	249.15	4.81	C15H22O3	Lipids and lipid‐like molecules	Prenol lipids	NA	0.00	2.21
(R)‐3‐Hydroxybutyric acid	103.04	1.06	C4H8O3	Organic acids and derivatives	Hydroxy acids and derivatives	C01089	0.00	2.13
(Z)‐9‐Heptadecenoic acid	267.23	7.70	C17H32O2	Lipids and lipid‐like molecules	Fatty acyls	C16536	0.00	1.33
delta.‐Nonalactone	157.12	3.59	C9H16O2	Organoheterocyclic compounds	Lactones	NA	0.01	1.36
1,1′‐[1,12‐Dodecanediylbis(oxy)]bisbenzene	355.26	4.35	C24H34O2	Benzenoids	Phenol ethers	NA	0.00	3.39
1,2‐Benzenediol bis(trimethylsilyl) ether	253.11	3.78	C12H22O2Si2	Benzenoids	Benzene and substituted derivatives	NA	0.00	1.55
1,2‐Dilinoleoyl‐sn‐glycero‐3‐phosphocholine	782.56	7.81	C44H80NO8P	Lipids and lipid‐like molecules s	Glycerophospholipid	NA	0.03	1.06
1,2‐Dipalmitoyl‐sn‐glycero‐3‐phospho‐(1′‐myo‐inositol)	809.51	7.76	C41H79O13P	Lipids and lipid‐like molecules s	Glycerophospholipid	NA	0.02	1.35
11.beta.,21‐Dihydroxy‐5.beta.‐pregnane‐3,20‐dione	349.23	3.63	C21H32O4	Lipids and lipid‐like molecules	Sterol lipids	C05475	0.00	3.15
12R‐Hydroxy‐5Z,8Z,10E,14Z‐eicosatetraenoic acid	319.23	5.68	C20H32O3	Lipids and lipid‐like molecules	Fatty acyls	NA	0.00	2.77
13′‐Carboxy‐alpha‐tocotrienol	453.30	5.82	C29H42O4	Lipids and lipid‐like molecules	Prenol lipids	NA	0.04	1.41
15,16‐DiHODE	311.22	5.17	C18H32O4	Lipids and lipid‐like molecules	Fatty acyls	NA	0.00	2.09
18‐Hydroxy‐11‐dehydrotetrahydrocorticosterone	363.22	4.04	C21H32O5	Lipids and lipid‐like molecules	Sterol lipids	NA	0.00	2.59
1H‐Indole‐4‐carboxaldehyde	146.06	3.48	C9H7NO	Organoheterocyclic compounds	Indoles and derivative	NA	0.00	1.59
1‐Hydroxyibuprofen	223.13	3.89	C13H18O3	Phenylpropanoids and polyketides	Phenylpropanoic acids	NA	0.00	1.32
1‐O‐Hexadecyl‐2‐O‐butanoyl‐sn‐glyceryl‐3‐phosphocholine	552.40	7.39	C28H58NO7P	Lipids and lipid‐like molecules	Glycerophospholipids	NA	0.00	1.45
2,3‐Diphenylpyrazine	233.11	4.06	C16H12N2	Organoheterocyclic compounds	Diazines	NA	0.02	1.08
2,4‐Octadiene	111.12	3.62	C8H14	Hydrocarbons	Unsaturated hydrocarbons	NA	0.03	1.24
2,6‐Dihydroxybenzoic acid	153.02	3.20	C7H6O4	Benzenoids	Benzene and substituted derivatives	NA	0.00	2.88
2,7,8‐Trimethyl‐2‐(.beta.‐carboxyethyl)‐6‐hydroxychroman	263.13	4.03	C15H20O4	NA	NA	NA	0.00	1.41
24‐Oxo‐1alpha,25‐dihydroxyvitamin D3	429.30	5.58	C27H42O4	Lipids and lipid‐like molecules	Sterol lipids	NA	0.00	2.72
2‐Butyl‐4‐methylphenol	163.11	4.39	C11H16O	Benzenoids	Benzene and substituted derivatives	NA	0.00	1.48
2‐Exo‐hydroxy‐1,8‐cineole	169.12	4.81	C10H18O2	Organoheterocyclic compounds	Oxanes	NA	0.00	1.12
2‐Indolecarboxylic acid	162.05	3.41	C9H7NO2	Organoheterocyclic compounds	Indoles and derivatives	NA	0.00	2.14
2‐Naphthaldehyde	155.05	1.02	C11H8O	Benzenoids	Naphthalenes	NA	0.01	1.51
3′,5’‐Cyclic AMP	330.06	2.72	C10H12N5O6P	Nucleosides, nucleotides, and analogueA		NA	0.00	1.64
3,5‐Di‐tert‐butyl‐4‐hydroxybenzoic acid	251.16	3.70	C15H22O3	Benzenoids	Benzene and substituted derivatives	NA	0.00	1.03
3‐Formyl‐6‐hydroxyindole	160.04	3.42	C9H7NO2	Organoheterocyclic compounds	Indoles and derivatives	NA	0.01	1.53
3‐Furancarboxylic acid, tetrahydro‐4‐methylene‐5‐oxo‐2‐propyl‐, (2R,3S)‐rel—	183.07	3.08	C9H12O4	NA	NA	NA	0.00	2.22
3‐Hydroxyoleylcarnitine	442.35	5.15	C25H47NO5	Lipids and lipid‐like molecules	Fatty acyls	NA	0	2.82
3‐Isobutylglutaric acid	187.10	3.15	C9H16O4	NA	NA	NA	0.01	1.14
3‐Methylazelaic acid	201.11	4.01	C10H18O4	Lipids and lipid‐like molecules	Fatty acyls	NA	0.01	1.45
3‐Hydroxyoleylcarnitine	442.35	5.15	C25H47NO5	Lipids and lipid‐like molecules	Fatty Acylsacyls	NA	0	2.82
3‐Isobutylglutaric acid	187.10	3.15	C9H16O4	NA	NA	NA	0.01	1.14
3‐Methylazelaic acid	201.11	4.01	C10H18O4	Lipids and lipid‐like molecules	Fatty Acylsacyls	NA	0.01	1.45
3‐Oxo‐1,4,11(13)‐eudesmatrien‐12‐oic acid	245.12	3.98	C15H18O3	Lipids and lipid‐like molecules	Prenol lipids	NA	0.01	1.17
4‐Pentenyl acetate	127.08	3.10	C7H12O2	Organic acids and derivatives	Carboxylic acids and derivatives	NA	0.00	1.79
5‐(2‐Furanyl)‐3,4‐dihydro‐2H‐pyrrole	134.06	2.93	C8H9NO	Organoheterocyclic compounds	Heteroaromatic compounds	NA	0.00	2.76
5‐Hydroxy‐6E,8Z,11Z,14Z‐eicosatetraenoic acid, 1,5‐lactone	303.23	5.68	C20H30O2	Lipids and lipid‐like molecules	Fatty acyls	NA	0.00	2.53
5‐Keto‐D‐gluconic acid	193.03	3.20	C6H10O7	Organic acids and derivatives	Hydroxy acids and derivatives	NA	0.01	1.31
6‐Azathymine	128.05	4.12	C4H5N3O2	NA	NA	NA	0	7.92
7alpha‐Hydroxypregnenolone	333.24	4.48	C21H32O3	Lipids and lipid‐like molecules	Sterol Lipidslipids	C18038	0.05	1.55
7′‐Carboxy‐alpha‐tocotrienol	345.21	4.56	C21H30O4	Organoheterocyclic compounds	Benzopyrans	NA	0.00	3.45
Acylcarnitine 10:0	316.25	3.99	C17H34NO4	Lipids and lipid‐like molecules	Fatty Acylsacyls	C02301	0.00	1.29
Acylcarnitine 12:1	342.26	4.18	C19H36NO4	Lipids and lipid‐like molecules	Fatty Acyls	C02301	0.02	1.13
Acylcarnitine 13:0	358.29	4.57	C20H40NO4	Lipids and lipid‐like molecules	Fatty acyls	C02301	0.00	1.29
Acylcarnitine 13:1	356.28	4.39	C20H38NO4	Lipids and lipid‐like molecules	Fatty acyls	C02301	0.02	1.30
Acylcarnitine 14:0	372.31	5.00	C21H42NO4	Lipids and lipid‐like molecules	Fatty acyls	C02301	0	1.89
Acylcarnitine 14:1	370.29	4.66	C21H40NO4	Lipids and lipid‐like molecules	Fatty acyls	C02301	0.00	1.53
Acylcarnitine 16:1	398.32	5.27	C23H44NO4	Lipids and lipid‐like molecules	Fatty acyls	C02301	0.00	1.93
Acylcarnitine 16:2	396.31	4.84	C23H42NO4	Lipids and lipid‐like molecules	Fatty acyls	C02301	0.00	1.29
Acylcarnitine 4:0	232.14	2.82	C11H22NO4	Lipids and lipid‐like molecules	Fatty acyls	C02301	0.00	1.70
Acylcarnitine 8:1	286.20	3.49	C15H28NO4	Lipids and lipid‐like molecules	Fatty acyls	C02301	0.00	1.20
Acylcarnitine 9:1	300.21	3.67	C16H30NO4	Lipids and lipid‐like molecules	Fatty acyls	C02301	0.04	1.06
all‐trans‐Retinoic acid	301.21	5.30	C20H28O2	Lipids and lipid‐like molecules	Prenol lipids	C00777	0.00	2.77
Atractylenolide III	247.13	4.01	C15H20O3	Lipids and lipid‐like molecules	Prenol lipids	NA	0.00	1.32
Atrazine‐desethyl	188.07	4.12	C6H10ClN5	Organoheterocyclic compounds	Triazines	C06559	0	7.49
Butralin	296.16	3.35	C14H21N3O4	NA	NA	C18582	0.00	1.70
Cholic acid	407.28	4.36	C24H40O5	Lipids and lipid‐like molecules	Steroids and steroid derivatives	C00695	0.00	3.12
cis‐3‐Octenyl propionate	185.15	3.63	C11H20O2	Lipids and lipid‐like molecules	Fatty acyls	NA	0.03	1.17
D‐(+)‐Glucosamine	180.10	0.81	C6H13NO5	Organic oxygen compounds	Organooxygen compounds	C00329	0.05	3.70
Deoxycholic acid	391.28	5.00	C24H40O4	Lipids and lipid‐like molecules	NA	C04483	0.02	2.21
Diethyl suberate	231.16	3.63	C12H22O4	NA	NA	NA	0.03	1.20
Dimethylhexa‐1,4‐diene	111.12	4.59	C8H14	Hydrocarbons	Unsaturated hydrocarbons	NA	0.03	1.05
DL‐Indole‐3‐lactic acid	204.07	3.20	C11H11NO3	Organoheterocyclic compounds	Indoles and derivatives	NA	0.00	1.10
D‐Pipecolinic acid	130.09	1.28	C6H11NO2	Organic acids and derivatives	Carboxylic acids and derivatives	NA	0.01	1.04
Equol 7‐O‐glucuronide	436.16	3.22	C21H22O9	Phenylpropanoids and polyketides	Isoflavonoids	NA	0.00	1.26
FAHFA 27:4; FAHFA (18:4/9:0)	431.31	5.34	C27H44O4	Lipids and lipid‐like molecules	Fatty acyls	NA	0.01	3.77
FAHFA 36:3; FAHFA (18:2/18:1)	559.47	7.47	C36H64O4	Lipids and lipid‐like molecules	Fatty acyls	NA	0.01	1.50
FAHFA 40:7; FAHFA (20:4/20:3)	607.47	7.31	C40H64O4	Lipids and lipid‐like molecules	Fatty Acyls	NA	0.00	1.17
Galanal A	317.21	5.30	C20H30O3	Organic oxygen compounds	Organooxygen compounds	NA	0.00	2.05
Gamma‐Linolenic acid	277.22	6.88	C18H30O2	Lipids and lipid‐like molecules	Fatty acyls	C06426	0.02	1.05
Glu‐Leu	261.15	4.02	C11H20N2O5	Organic acids and derivatives	Carboxylic acids and derivatives	NA	0.00	1.63
Goshuyic acid	223.17	5.83	C14H24O2	Lipids and lipid‐like molecules	Fatty acyls	NA	0.01	1.04
Hexanoyl‐L‐carnitine	260.18	3.29	C13H25NO4	Lipids and lipid‐like molecules	Fatty acyls	NA	0.00	1.19
Hippuric acid	178.05	2.94	C9H9NO3	Benzenoids	Benzene and substituted derivatives	C01586	0.00	2.67
Hirsutine	369.22	3.83	C22H28N2O3	Alkaloids and derivatives	NA	NA	0.00	1.97
Hydrocinnamic acid	149.06	3.86	C9H10O2	Phenylpropanoids and polyketides	Phenylpropanoic acids	C05629	0.00	1.77
Imiquimod	239.13	3.89	C14H16N4	Organoheterocyclic compounds	Quinolines and derivatives	NA	0.00	1.52
Isobutylangelate	155.11	3.22	C9H16O2	Lipids and lipid‐like molecules	Fatty Acyls	NA	0.02	1.30
Isolongifolene, 4,5,9,10‐dehydro—	199.14	3.46	C15H20	Hydrocarbons	Polycyclic hydrocarbons	NA	0.03	1.53
Kessyl glycol	253.18	4.31	C15H26O3	Organoheterocyclic compounds	Oxepanes	NA	0.00	2.02
L‐3‐Phenyllactic acid	165.06	3.38	C9H10O3	Phenylpropanoids and polyketides	Phenylpropanoic acids	C05607	0.01	2.65
Lauroyl‐L‐carnitine	344.28	4.42	C19H37NO4	Lipids and lipid‐like molecules	Fatty acyls	NA	0.00	1.20
inoleoylcarnitine	424.34	5.55	C25H45NO4	Lipids and lipid‐like molecules	Fatty acyls	NA	0.03	1.96
LsoPC 16:2	492.30	4.67	C24H46NO7P	Lipids and lipid‐like molecules	Glycerophospholipids	C04230	0.00	1.37
LysoPC 17:1	566.34	5.32	C25H50NO7P	Lipids and lipid‐like molecules	Glycerophospholipids	C04230	0.00	1.07
LysoPC 18:2	578.34	5.20	C26H50NO7P	Lipids and lipid‐like molecules	Glycerophospholipids	C04230	0.00	1.13
LysoPC 20:2	606.38	5.92	C28H54NO7P	Lipids and lipid‐like molecules	Glycerophospholipids	C04230	0.00	2.00
LysoPC 20:3	604.36	5.48	C28H52NO7P	Lipids and lipid‐like molecules	Glycerophospholipids	C04230	0.01	2.69
LysoPC 20:5	600.33	4.86	C28H48NO7P	Lipids and lipid‐like molecules	Glycerophospholipids	C04230	0.00	1.38
LysoPC 22:4	630.38	5.79	C30H54NO7P	Lipids and lipid‐like molecules	Glycerophospholipids	C04230	0.00	1.18
LysoPC 22:5	628.36	5.35	C30H52NO7P	Lipids and lipid‐like molecules	Glycerophospholipids	C04230	0.00	1.00
N,N‐dimethylindoliumolate	176.07	3.46	C10H11NO2	Organoheterocyclic compounds	Indoles and derivatives	NA	0	6.08
N‐Desmethylmirtazapine	252.17	3.81	C16H17N3	NA	NA	NA	0.01	1.21
Norharman	169.08	3.53	C11H8N2	Organoheterocyclic compounds	Indoles and derivatives	C20157	0.00	1.29
Norharmane	169.07	3.10	C11H8N2	Organoheterocyclic compounds	Indoles and derivatives	C20157	0.05	1.42
N‐Stearoyltaurine	390.27	5.54	C20H41NO4S	NA	NA	NA	0.00	1.10
O‐Acetyl‐L‐carnitine	204.12	1.20	C9H18NO4	Lipids and lipid‐like molecules	Fatty acyls	C02571	0.01	1.03
Octadecadienoate	279.23	7.47	C18H32O2	Lipids and lipid‐like molecules	Fatty acyls	NA	0.01	1.06
Octanoylcarnitine	288.21	3.63	C15H29NO4	Lipids and lipid‐like molecules	Fatty acyls	C02838	0.00	1.02
Oleic acid	281.25	8.18	C18H34O2	Lipids and lipid‐like molecules	Fatty acyls	C00712	0.00	1.08
Oleoyl‐L‐carnitine	426.35	6.16	C25H47NO4	Lipids and lipid‐like molecules	Fatty acyls	NA	0.00	2.21
Palmitelaidic acid	253.22	7.22	C16H30O2	Lipids and lipid‐like molecules	Fatty Acyls	NA	0.00	1.51
Palmitoyl sphingomyelin	703.57	7.81	C39H79N2O6P	NA	NA	NA	0.01	1.32
Palmitoylcarnitine	400.34	5.88	C23H45NO4	Lipids and lipid‐like molecules	Fatty Acyls	C02990	0	1.80
PC 34:2; PC (16:0/18:2)	816.57	9.63	C42H80NO8P	Lipids and lipid‐like molecules	Glycerophospholipids	C00157	0.00	1.96
PC 36:4; PC (16:0/20:4)	840.57	5.68	C44H80NO8P	Lipids and lipid‐like molecules	Glycerophospholipids	C00157	0.00	2.16
PC 38:4; PC (18:0/20:4)	868.60	9.63	C46H84NO8P	Lipids and lipid‐like molecules	Glycerophospholipids	C00157	0.00	1.10
PC 38:6; PC (16:0/22:6)	864.57	9.63	C46H80NO8P	Lipids and lipid‐like molecules	Glycerophospholipids	C00157	0.01	1.41
PC (16:0/16:1 (9Z))	732.55	9.65	C40H78NO8P	Lipids and lipid‐like molecules	Glycerophospholipids	C00157	0.00	1.72
PC (18:2 (9Z,12Z)/20:3 (5Z,8Z,11Z))	808.58	4.09	C46H82NO8P	Lipids and lipid‐like molecules	Glycerophospholipids	C00157	0.00	1.15
p‐Cresol sulfate	187.01	3.31	C7H8O4S	Organic acids and derivatives	Organic sulfuric acids and derivatives	NA	0.00	2.84
Pelargonic acid	157.12	4.69	C9H18O2	Lipids and lipid‐like molecules	Fatty acyls	C01601	0.04	1.24
Phenol	93.03	3.05	C6H6O	Benzenoids	Phenols	C00146	0.02	1.22
Phenol sulphate	172.99	3.05	C6H6O4S	Organic acids and derivatives	Organic sulfuric acids and derivatives	C00850	0.01	1.55
Phenyl glucuronide	269.07	2.75	C12H14O7	NA	NA	NA	0.02	1.62
Physoperuvine	200.13	3.58	C8H15NO	Alkaloids and derivatives	Tropane alkaloids	C10864	0.01	1.39
PI 33:2; PI (15:0/18:2)	819.50	7.44	C42H77O13P	Lipids and lipid‐like molecules	Glycerophospholipids	C01194	0.00	2.12
PI 34:2; PI (16:0/18:2)	833.52	7.59	C43H79O13P	Lipids and lipid‐like molecules	Glycerophospholipids	C01194	0.02	1.12
PI 35:2; PI (17:0/18:2)	847.53	7.72	C44H81O13P	Lipids and lipid‐like molecules	Glycerophospholipids	C01194	0.00	2.22
PI 35:4; PI (15:0/20:4)	843.50	7.40	C44H77O13P	Lipids and lipid‐like molecules	Glycerophospholipids	C01194	0.00	1.74
PI 36:1; PI (18:0/18:1)	863.56	8.08	C45H85O13P	Lipids and lipid‐like molecules	Glycerophospholipids	C01194	0.00	2.17
PI 36:2; PI (18:0/18:2)	861.55	7.85	C45H83O13P	Lipids and lipid‐like molecules	Glycerophospholipids	C01194	0.00	1.19
PI 37:4; PI (17:0/20:4)	871.53	7.69	C46H81O13P	Lipids and lipid‐like molecules	Glycerophospholipids	C01194	0.04	1.50
PI 38:2; PI (18:0/20:2)	889.58	8.12	C47H87O13P	Lipids and lipid‐like molecules	Glycerophospholipids	C01194	0.00	2.20
PI 38:5; PI (18:1/20:4)	883.53	7.58	C47H81O13P	Lipids and lipid‐like molecules	Glycerophospholipids	C01194	0.00	1.71
PI 39:4; PI (19:0/20:4)	899.56	7.93	C48H85O13P	Lipids and lipid‐like molecules	Glycerophospholipids	C01194	0.03	1.59
PI 39:5; PI (17:0/22:5)	897.55	7.70	C48H83O13P	Lipids and lipid‐like molecules	Glycerophospholipids	C01194	0.01	2.03
PI 39:6; PI (17:0/22:6)	895.53	7.59	C48H81O13P	Lipids and lipid‐like molecules	Glycerophospholipids	C01194	0.00	2.30
PI 40:4; PI (18:0/22:4)	913.58	8.00	C49H87O13P	Lipids and lipid‐like molecules	Glycerophospholipids	C01194	0.01	1.92
PI 40:7; PI (18:1/22:6)	907.53	7.52	C49H81O13P	Lipids and lipid‐like molecules	Glycerophospholipids	C01194	0.01	1.65
Plasmenyl‐PE 37:4; PE (P‐17:0/20:4)	736.53	8.47	C42H76NO7P	Lipids and lipid‐like molecules	Glycerophospholipids	C04756	0.02	3.20
Plasmenyl‐PE 40:5; PE (P‐18:0/22:5)	776.56	8.49	C45H80NO7P	Lipids and lipid‐like molecules	Glycerophospholipids	C04756	0.04	1.43
Plasmenyl‐PE 40:6; PE (P‐18:0/22:6)	774.54	8.49	C45H78NO7P	Lipids and lipid‐like molecules	Glycerophospholipids	C04756	0.04	1.48
Prenyl caproate	185.15	4.59	C11H20O2	Lipids and lipid‐like molecules	Fatty acyls	C13422	0.03	1.06
Quinaldic acid	174.05	3.09	C10H7NO2	Organoheterocyclic compounds	Quinolines and derivatives	C06325	0	7.35
Retinyl ester	301.22	5.68	C20H30O2	Lipids and lipid‐like molecules	Prenol lipids	C02075	0.00	2.75
Salicylic acid	137.02	3.07	C7H6O3	Benzenoids	Benzene and substituted derivatives	C00805	0.00	2.15
Santene hydrate	139.11	4.59	C9H16O	Lipids and lipid‐like molecules	Prenol lipids	NA	0.03	1.11
Sayanedin	299.09	3.46	C17H14O5	Phenylpropanoids and polyketides	Isoflavonoids	C10527	0.02	2.10
Sciadonic acid	305.25	7.93	C20H34O2	Lipids and lipid‐like molecules	Fatty acyls	NA	0.00	1.61
Sulfameter	281.07	3.56	C11H12N4O3S	NA	NA	NA	0.00	3.61
Tamoxifen	372.23	2.94	C26H29NO	Phenylpropanoids and polyketides	Stilbenes	C07108	0.00	2.30
Taurodeoxycholic acid	498.29	3.88	C26H45NO6S	Lipids and lipid‐like molecules	Sterol Lipids	C05463	0.02	1.90
Trachelanthine	302.19	3.17	C15H27NO5	Organoheterocyclic compounds	Indoles and derivatives	NA	0.00	1.61
Trilobinone	315.19	4.66	C20H28O3	Lipids and lipid‐like molecules	Prenol lipids	NA	0.01	1.40
Ursocholic acid	467.30	4.07	C24H40O5	Lipids and lipid‐like molecules	Sterol lipids	C17644	0.00	2.54
Yucalexin A19	317.21	4.99	C20H30O3	Lipids and lipid‐like molecules	Prenol lipids	NA	0.02	1.64

In addition, we annotated all the differential metabolites in Table 2 on the basis of the KEGG database and performed pathway analysis for these metabolites by using metabolite analysts. It was found that the most prominent metabolic pathways were glycerophospholipid metabolism and steroid hormone biosynthesis (Figure 6E). In conclusion, these results suggest that DG may treat osteoporosis by affecting lipid metabolism in OVX rats.

### 3.5 Analysis of Serum Differential Metabolites in FL‐Treated OVX Rats

**FIGURE 7:**
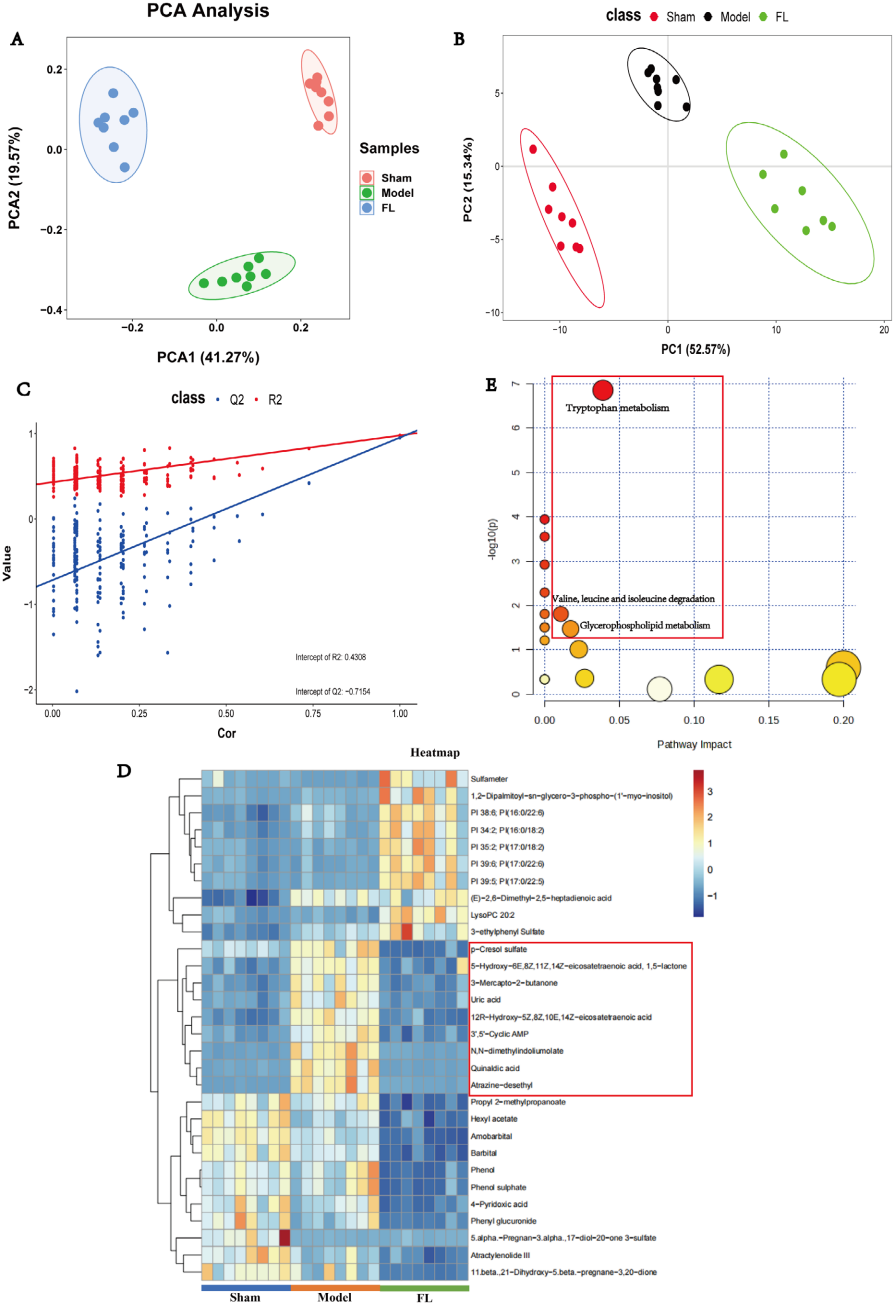
Metabolomic analysis of FL treatment, model, and sham groups. (A) PCA analysis of FL; (B, C) PLS‐DA of FL; (D) heat map and cluster analysis of differential metabolites. (E) Functional enrichment analysis of FL differential metabolites.

The results of the PCA and PLS‐DA analyses demonstrated significant within‐group clustering between the FL, sham, and model groups, indicating notable differences in metabolites between these three groups (Figure 7A–C). All differential metabolites, identified by PLS‐DA analysis, are presented in Table 3. Furthermore, the top 30 significant differential metabolites between the sham, model, and FL groups were shown in a clustered heatmap (Figure 7D). Among them, we found nine differential metabolites that were up‐regulated following modelling while down‐regulated after FL treatment, namely p‐cresol sulfate, 5‐hydroxy‐6E,8Z,11Z,14Z‐eicosatetraenoic acid, 1,5‐lactone, 3‐mercapto‐2‐butanone, uric acid, 12R‐hydroxy‐5Z,8Z,10E,14‐Zeicosatetraenoic acid, 3′,5′‐cyclic AMP, N,N‐dimethylindoliumolate, quinaldic acid, and atrazine‐desethyl. These nine metabolites are mainly related to amino acid metabolism and lipid metabolism. Pathway impact analysis of all differential metabolites in Table 3 showed that the most significant metabolic pathways were tryptophan metabolism, degradation of valine, leucine, and isoleucine, and glycerophospholipid metabolism (Figure 7E). Taken together, these results suggest that FL may treat osteoporosis by affecting lipid and amino acid metabolisms in OVX rats.

**TABLE 3:** All the differential metabolites screened among Sham, Model and FL groups (VIP > 1; *p* < 0.05).

Name	MZ	RT	Formula	SuperClass	Class	KEGG	*p*	VIP
(1R)‐Chrysanthemolactone	186.16	3.62	C10H16O2	Organoheterocyclic compounds	Lactones	NA	0.03	1.17
(2E)‐Decenoyl‐ACP	128.07	1.28	C6H11NO2	Organic acids and derivatives	Carboxylic acids and derivatives	C03969	0.00	1.24
(3beta, 8beta)‐3‐Hydroxy‐7(11)‐eremophilen‐12,8‐olide	249.15	4.81	C15H22O3	Lipids and lipid‐like molecules	Prenol lipids	NA	0.00	2.21
(R)‐3‐Hydroxybutyric acid	103.04	1.06	C4H8O3	Organic acids and derivatives	Hydroxy acids and derivatives	C01089	0.00	2.13
(Z)‐9‐Heptadecenoic acid	267.23	7.70	C17H32O2	Lipids and lipid‐like molecules	Fatty Acyls	C16536	0.00	1.33
.delta.‐Nonalactone	157.12	3.59	C9H16O2	Organoheterocyclic compounds	Lactones	NA	0.01	1.36
1,1′‐[1,12‐Dodecanediylbis(oxy)]bisbenzene	355.26	4.35	C24H34O2	Benzenoids	Phenol ethers	NA	0.00	3.39
1,2‐Benzenediol bis(trimethylsilyl) ether	253.11	3.78	C12H22O2Si2	Benzenoids	Benzene and substituted derivatives	NA	0.00	1.55
1,2‐Dilinoleoyl‐sn‐glycero‐3‐phosphocholine	782.56	7.81	C44H80NO8P	Lipids and lipid‐like molecules	Glycerophospholipids	NA	0.03	1.06
1,2‐Dipalmitoyl‐sn‐glycero‐3‐phospho‐(1′‐myo‐inositol)	809.51	7.76	C41H79O13P	Lipids and lipid‐like molecules	Glycerophospholipids	NA	0.02	1.35
11.beta.,21‐Dihydroxy‐5.beta.‐pregnane‐3,20‐dione	349.23	3.63	C21H32O4	Lipids and lipid‐like molecules	Sterol Lipids	C05475	0.00	3.15
12R‐Hydroxy‐5Z,8Z,10E,14Z‐eicosatetraenoic acid	319.23	5.68	C20H32O3	Lipids and lipid‐like molecules	Fatty Acyls	NA	0.00	2.77
13′‐Carboxy‐alpha‐tocotrienol	453.30	5.82	C29H42O4	Lipids and lipid‐like molecules	Prenol lipids	NA	0.04	1.41
15,16‐DiHODE	311.22	5.17	C18H32O4	Lipids and lipid‐like molecules	Fatty Acyls	NA	0.00	2.09
18‐Hydroxy‐11‐dehydrotetrahydrocorticosterone	363.22	4.04	C21H32O5	Lipids and lipid‐like molecules	Sterol Lipids	NA	0.00	2.59
1H‐Indole‐4‐carboxaldehyde	146.06	3.48	C9H7NO	Organoheterocyclic compounds	Indoles and derivatives	NA	0.00	1.59
1‐Hydroxyibuprofen	223.13	3.89	C13H18O3	Phenylpropanoids and polyketides	Phenylpropanoic acids	NA	0.00	1.32
1‐O‐Hexadecyl‐2‐O‐butanoyl‐sn‐glyceryl‐3‐phosphocholine	552.40	7.39	C28H58NO7P	Lipids and lipid‐like molecules	Glycerophospholipids	NA	0.00	1.45
2,3‐Diphenylpyrazine	233.11	4.06	C16H12N2	Organoheterocyclic compounds	Diazines	NA	0.02	1.08
2,4‐Octadiene	111.12	3.62	C8H14	Hydrocarbons	Unsaturated hydrocarbons	NA	0.03	1.24
2,6‐Dihydroxybenzoic acid	153.02	3.20	C7H6O4	Benzenoids	Benzene and substituted derivatives	NA	0.00	2.88
2,7,8‐Trimethyl‐2‐(.beta.‐carboxyethyl)‐6‐hydroxychroman	263.13	4.03	C15H20O4	NA	NA	NA	0.00	1.41
24‐Oxo‐1alpha,25‐dihydroxyvitamin D3	429.30	5.58	C27H42O4	Lipids and lipid‐like molecules	Sterol Lipids	NA	0.00	2.72
2‐Butyl‐4‐methylphenol	163.11	4.39	C11H16O	Benzenoids	Benzene and substituted derivatives	NA	0.00	1.48
2‐Exo‐hydroxy‐1,8‐cineole	169.12	4.81	C10H18O2	Organoheterocyclic compounds	Oxanes	NA	0.00	1.12
2‐Indolecarboxylic acid	162.05	3.41	C9H7NO2	Organoheterocyclic compounds	Indoles and derivatives	NA	0.00	2.14
2‐Naphthaldehyde	155.05	1.02	C11H8O	Benzenoids	Naphthalenes	NA	0.01	1.51
3′,5′‐Cyclic AMP	330.06	2.72	C10H12N5O6P	Nucleosides, nucleotides, and analogue A	NA	0.00	1.64	
3,5‐Di‐tert‐butyl‐4‐hydroxybenzoic acid	251.16	3.70	C15H22O3	Benzenoids	Benzene and substituted derivatives	NA	0.00	1.03
3‐Formyl‐6‐hydroxyindole	160.04	3.42	C9H7NO2	Organoheterocyclic compounds	Indoles and derivatives	NA	0.01	1.53
3‐Furancarboxylic acid, tetrahydro‐4‐methylene‐5‐oxo‐2‐propyl‐, (2R,3S)‐rel—	183.07	3.08	C9H12O4	NA	NA	NA	0.00	2.22
3‐Hydroxyoleylcarnitine	442.35	5.15	C25H47NO5	Lipids and lipid‐like molecules	Fatty Acyls	NA	0	2.82
3‐Isobutylglutaric acid	187.10	3.15	C9H16O4	NA	NA	NA	0.01	1.14
3‐Methylazelaic acid	201.11	4.01	C10H18O4	Lipids and lipid‐like molecules	Fatty Acyls	NA	0.01	1.45
3‐Oxo‐1,4,11(13)‐eudesmatrien‐12‐oic acid	245.12	3.98	C15H18O3	Lipids and lipid‐like molecules	Prenol lipids	NA	0.01	1.17
4‐Pentenyl acetate	127.08	3.10	C7H12O2	Organic acids and derivatives	Carboxylic acids and derivatives	NA	0.00	1.79
5‐(2‐Furanyl)‐3,4‐dihydro‐2H‐pyrrole	134.06	2.93	C8H9NO	Organoheterocyclic compounds	Heteroaromatic compounds	NA	0.00	2.76
5‐Hydroxy‐6E,8Z,11Z,14Z‐eicosatetraenoic acid, 1,5‐lactone	303.23	5.68	C20H30O2	Lipids and lipid‐like molecules	Fatty Acyls	NA	0.00	2.53
5‐Keto‐D‐gluconic acid	193.03	3.20	C6H10O7	Organic acids and derivatives	Hydroxy acids and derivatives	NA	0.01	1.31
6‐Azathymine	128.05	4.12	C4H5N3O2	NA	NA	NA	0	7.92
7alpha‐Hydroxypregnenolone	333.24	4.48	C21H32O3	Lipids and lipid‐like molecules	Sterol Lipids	C18038	0.05	1.55
7′‐Carboxy‐alpha‐tocotrienol	345.21	4.56	C21H30O4	Organoheterocyclic compounds	Benzopyrans	NA	0.00	3.45
Acylcarnitine 10:0	316.25	3.99	C17H34NO4	Lipids and lipid‐like molecules	Fatty Acyls	C02301	0.00	1.29
Acylcarnitine 12:1	342.26	4.18	C19H36NO4	Lipids and lipid‐like molecules	Fatty Acyls	C02301	0.02	1.13
Acylcarnitine 13:0	358.29	4.57	C20H40NO4	Lipids and lipid‐like molecules	Fatty Acyls	C02301	0.00	1.29
Acylcarnitine 13:1	356.28	4.39	C20H38NO4	Lipids and lipid‐like molecules	Fatty Acyls	C02301	0.02	1.30
Acylcarnitine 14:0	372.31	5.00	C21H42NO4	Lipids and lipid‐like molecules	Fatty Acyls	C02301	0	1.89
Acylcarnitine 14:1	370.29	4.66	C21H40NO4	Lipids and lipid‐like molecules	Fatty Acyls	C02301	0.00	1.53
Acylcarnitine 16:1	398.32	5.27	C23H44NO4	Lipids and lipid‐like molecules	Fatty Acyls	C02301	0.00	1.93
Acylcarnitine 16:2	396.31	4.84	C23H42NO4	Lipids and lipid‐like molecules	Fatty Acyls	C02301	0.00	1.29
Acylcarnitine 4:0	232.14	2.82	C11H22NO4	Lipids and lipid‐like molecules	Fatty Acyls	C02301	0.00	1.70
Acylcarnitine 8:1	286.20	3.49	C15H28NO4	Lipids and lipid‐like molecules	Fatty Acyls	C02301	0.00	1.20
Acylcarnitine 9:1	300.21	3.67	C16H30NO4	Lipids and lipid‐like molecules	Fatty Acyls	C02301	0.04	1.06
all‐trans‐Retinoic acid	301.21	5.30	C20H28O2	Lipids and lipid‐like molecules	Prenol lipids	C00777	0.00	2.77
Atractylenolide III	247.13	4.01	C15H20O3	Lipids and lipid‐like molecules	Prenol lipids	NA	0.00	1.32
Atrazine‐desethyl	188.07	4.12	C6H10ClN5	Organoheterocyclic compounds	Triazines	C06559	0	7.49
Butralin	296.16	3.35	C14H21N3O4	NA	NA	C18582	0.00	1.70
Cholic acid	407.28	4.36	C24H40O5	Lipids and lipid‐like molecules	Steroids and steroid derivatives	C00695	0.00	3.12
cis‐3‐Octenyl propionate	185.15	3.63	C11H20O2	Lipids and lipid‐like molecules	Fatty Acyls	NA	0.03	1.17
D‐(+)‐Glucosamine	180.10	0.81	C6H13NO5	Organic oxygen compounds	Organooxygen compounds	C00329	0.05	3.70
Deoxycholic acid	391.28	5.00	C24H40O4	Lipids and lipid‐like molecules	NA	C04483	0.02	2.21
Diethyl suberate	231.16	3.63	C12H22O4	NA	NA	NA	0.03	1.20
Dimethylhexa‐1,4‐diene	111.12	4.59	C8H14	Hydrocarbons	Unsaturated hydrocarbons	NA	0.03	1.05
DL‐Indole‐3‐lactic acid	204.07	3.20	C11H11NO3	Organoheterocyclic compounds	Indoles and derivatives	NA	0.00	1.10
D‐Pipecolinic acid	130.09	1.28	C6H11NO2	Organic acids and derivatives	Carboxylic acids and derivatives	NA	0.01	1.04
Equol 7‐O‐glucuronide	436.16	3.22	C21H22O9	Phenylpropanoids and polyketides	Isoflavonoids	NA	0.00	1.26
FAHFA 27:4; FAHFA (18:4/9:0)	431.31	5.34	C27H44O4	Lipids and lipid‐like molecules	Fatty Acyls	NA	0.01	3.77
FAHFA 36:3; FAHFA (18:2/18:1)	559.47	7.47	C36H64O4	Lipids and lipid‐like molecules	Fatty Acyls	NA	0.01	1.50
FAHFA 40:7; FAHFA (20:4/20:3)	607.47	7.31	C40H64O4	Lipids and lipid‐like molecules	Fatty Acyls	NA	0.00	1.17
Galanal A	317.21	5.30	C20H30O3	Organic oxygen compounds	Organooxygen compounds	NA	0.00	2.05
Gamma‐Linolenic acid	277.22	6.88	C18H30O2	Lipids and lipid‐like molecules	Fatty Acyls	C06426	0.02	1.05
Glu‐Leu	261.15	4.02	C11H20N2O5	Organic acids and derivatives	Carboxylic acids and derivatives	NA	0.00	1.63
Goshuyic acid	223.17	5.83	C14H24O2	Lipids and lipid‐like molecules	Fatty Acyls	NA	0.01	1.04
Hexanoyl‐L‐carnitine	260.18	3.29	C13H25NO4	Lipids and lipid‐like molecules	Fatty Acyls	NA	0.00	1.19
Hippuric acid	178.05	2.94	C9H9NO3	Benzenoids	Benzene and substituted derivatives	C01586	0.00	2.67
Hirsutine	369.22	3.83	C22H28N2O3	Alkaloids and derivatives	NA	NA	0.00	1.97
Hydrocinnamic acid	149.06	3.86	C9H10O2	Phenylpropanoids and polyketides	Phenylpropanoic acids	C05629	0.00	1.77
Imiquimod	239.13	3.89	C14H16N4	Organoheterocyclic compounds	Quinolines and derivatives	NA	0.00	1.52
Isobutylangelate	155.11	3.22	C9H16O2	Lipids and lipid‐like molecules	Fatty Acyls	NA	0.02	1.30
Isolongifolene, 4,5,9,10‐dehydro—	199.14	3.46	C15H20	Hydrocarbons	Polycyclic hydrocarbons	NA	0.03	1.53
Kessyl glycol	253.18	4.31	C15H26O3	Organoheterocyclic compounds	Oxepanes	NA	0.00	2.02
L‐3‐Phenyllactic acid	165.06	3.38	C9H10O3	Phenylpropanoids and polyketides	Phenylpropanoic acids	C05607	0.01	2.65
Lauroyl‐L‐carnitine	344.28	4.42	C19H37NO4	Lipids and lipid‐like molecules	Fatty Acyls	NA	0.00	1.20
Linoleoylcarnitine	424.34	5.55	C25H45NO4	Lipids and lipid‐like molecules	Fatty Acyls	NA	0.03	1.96
LysoPC 16:2	492.30	4.67	C24H46NO7P	Lipids and lipid‐like molecules	Glycerophospholipids	C04230	0.00	1.37
LysoPC 17:1	566.34	5.32	C25H50NO7P	Lipids and lipid‐like molecules	Glycerophospholipids	C04230	0.00	1.07
LysoPC 18:2	578.34	5.20	C26H50NO7P	Lipids and lipid‐like molecules	Glycerophospholipids	C04230	0.00	1.13
LysoPC 20:2	606.38	5.92	C28H54NO7P	Lipids and lipid‐like molecules	Glycerophospholipids	C04230	0.00	2.00
LysoPC 20:3	604.36	5.48	C28H52NO7P	Lipids and lipid‐like molecules	Glycerophospholipids	C04230	0.01	2.69
LysoPC 20:5	600.33	4.86	C28H48NO7P	Lipids and lipid‐like molecules	Glycerophospholipids	C04230	0.00	1.38
LysoPC 22:4	630.38	5.79	C30H54NO7P	Lipids and lipid‐like molecules	Glycerophospholipids	C04230	0.00	1.18
LysoPC 22:5	628.36	5.35	C30H52NO7P	Lipids and lipid‐like molecules	Glycerophospholipids	C04230	0.00	1.00
N,N‐dimethylindoliumolate	176.07	3.46	C10H11NO2	Organoheterocyclic compounds	Indoles and derivatives	NA	0	6.08
N‐Desmethylmirtazapine	252.17	3.81	C16H17N3	NA	NA	NA	0.01	1.21
Norharman	169.08	3.53	C11H8N2	Organoheterocyclic compounds	Indoles and derivatives	C20157	0.00	1.29
Norharmane	169.07	3.10	C11H8N2	Organoheterocyclic compounds	Indoles and derivatives	C20157	0.05	1.42
N‐Stearoyltaurine	390.27	5.54	C20H41NO4S	NA	NA	NA	0.00	1.10
O‐Acetyl‐L‐carnitine	204.12	1.20	C9H18NO4	Lipids and lipid‐like molecules	Fatty Acyls	C02571	0.01	1.03
Octadecadienoate	279.23	7.47	C18H32O2	Lipids and lipid‐like molecules	Fatty Acyls	NA	0.01	1.06
Octanoylcarnitine	288.21	3.63	C15H29NO4	Lipids and lipid‐like molecules	Fatty Acyls	C02838	0.00	1.02
Oleic acid	281.25	8.18	C18H34O2	Lipids and lipid‐like molecules	Fatty Acyls	C00712	0.00	1.08
Oleoyl‐L‐carnitine	426.35	6.16	C25H47NO4	Lipids and lipid‐like molecules	Fatty Acyls	NA	0.00	2.21
Palmitelaidic acid	253.22	7.22	C16H30O2	Lipids and lipid‐like molecules	Fatty Acyls	NA	0.00	1.51
Palmitoyl sphingomyelin	703.57	7.81	C39H79N2O6P	NA	NA	NA	0.01	1.32
Palmitoylcarnitine	400.34	5.88	C23H45NO4	Lipids and lipid‐like molecules	Fatty Acyls	C02990	0	1.80
PC 34:2; PC (16:0/18:2)	816.57	9.63	C42H80NO8P	Lipids and lipid‐like molecules	Glycerophospholipids	C00157	0.00	1.96
PC 36:4; PC (16:0/20:4)	840.57	5.68	C44H80NO8P	Lipids and lipid‐like molecules	Glycerophospholipids	C00157	0.00	2.16
PC 38:4; PC (18:0/20:4)	868.60	9.63	C46H84NO8P	Lipids and lipid‐like molecules	Glycerophospholipids	C00157	0.00	1.10
PC 38:6; PC (16:0/22:6)	864.57	9.63	C46H80NO8P	Lipids and lipid‐like molecules	Glycerophospholipids	C00157	0.01	1.41
PC (16:0/16:1 (9Z))	732.55	9.65	C40H78NO8P	Lipids and lipid‐like molecules	Glycerophospholipids	C00157	0.00	1.72
PC (18:2 (9Z,12Z)/20:3 (5Z,8Z,11Z))	808.58	4.09	C46H82NO8P	Lipids and lipid‐like molecules	Glycerophospholipids	C00157	0.00	1.15
p‐Cresol sulfate	187.01	3.31	C7H8O4S	Organic acids and derivatives	Organic sulfuric acids and derivatives	NA	0.00	2.84
Pelargonic acid	157.12	4.69	C9H18O2	Lipids and lipid‐like molecules	Fatty Acyls	C01601	0.04	1.24
Phenol	93.03	3.05	C6H6O	Benzenoids	Phenols	C00146	0.02	1.22
Phenol sulphate	172.99	3.05	C6H6O4S	Organic acids and derivatives	Organic sulfuric acids and derivatives	C00850	0.01	1.55
Phenyl glucuronide	269.07	2.75	C12H14O7	NA	NA	NA	0.02	1.62
Physoperuvine	200.13	3.58	C8H15NO	Alkaloids and derivatives	Tropane alkaloids	C10864	0.01	1.39
PI 33:2; PI (15:0/18:2)	819.50	7.44	C42H77O13P	Lipids and lipid‐like molecules	Glycerophospholipids	C01194	0.00	2.12
PI 34:2; PI (16:0/18:2)	833.52	7.59	C43H79O13P	Lipids and lipid‐like molecules	Glycerophospholipids	C01194	0.02	1.12
PI 35:2; PI (17:0/18:2)	847.53	7.72	C44H81O13P	Lipids and lipid‐like molecules	Glycerophospholipids	C01194	0.00	2.22
PI 35:4; PI (15:0/20:4)	843.50	7.40	C44H77O13P	Lipids and lipid‐like molecules	Glycerophospholipids	C01194	0.00	1.74
PI 36:1; PI (18:0/18:1)	863.56	8.08	C45H85O13P	Lipids and lipid‐like molecules	Glycerophospholipids	C01194	0.00	2.17
PI 36:2; PI (18:0/18:2)	861.55	7.85	C45H83O13P	Lipids and lipid‐like molecules	Glycerophospholipids	C01194	0.00	1.19
PI 37:4; PI (17:0/20:4)	871.53	7.69	C46H81O13P	Lipids and lipid‐like molecules	Glycerophospholipids	C01194	0.04	1.50
PI 38:2; PI (18:0/20:2)	889.58	8.12	C47H87O13P	Lipids and lipid‐like molecules	Glycerophospholipids	C01194	0.00	2.20
PI 38:5; PI (18:1/20:4)	883.53	7.58	C47H81O13P	Lipids and lipid‐like molecules	Glycerophospholipids	C01194	0.00	1.71
PI 39:4; PI (19:0/20:4)	899.56	7.93	C48H85O13P	Lipids and lipid‐like molecules	Glycerophospholipids	C01194	0.03	1.59
PI 39:5; PI (17:0/22:5)	897.55	7.70	C48H83O13P	Lipids and lipid‐like molecules	Glycerophospholipids	C01194	0.01	2.03
PI 39:6; PI (17:0/22:6)	895.53	7.59	C48H81O13P	Lipids and lipid‐like molecules	Glycerophospholipids	C01194	0.00	2.30
PI 40:4; PI (18:0/22:4)	913.58	8.00	C49H87O13P	Lipids and lipid‐like molecules	Glycerophospholipids	C01194	0.01	1.92
PI 40:7; PI (18:1/22:6)	907.53	7.52	C49H81O13P	Lipids and lipid‐like molecules	Glycerophospholipids	C01194	0.01	1.65
Plasmenyl‐PE 37:4; PE (P‐17:0/20:4)	736.53	8.47	C42H76NO7P	Lipids and lipid‐like molecules	Glycerophospholipids	C04756	0.02	3.20
Plasmenyl‐PE 40:5; PE (P‐18:0/22:5)	776.56	8.49	C45H80NO7P	Lipids and lipid‐like molecules	Glycerophospholipids	C04756	0.04	1.43
Plasmenyl‐PE 40:6; PE (P‐18:0/22:6)	774.54	8.49	C45H78NO7P	Lipids and lipid‐like molecules	Glycerophospholipids	C04756	0.04	1.48
Prenyl caproate	185.15	4.59	C11H20O2	Lipids and lipid‐like molecules	Fatty Acyls	C13422	0.03	1.06
Quinaldic acid	174.05	3.09	C10H7NO2	Organoheterocyclic compounds	Quinolines and derivatives	C06325	0	7.35
Retinyl ester	301.22	5.68	C20H30O2	Lipids and lipid‐like molecules	Prenol lipids	C02075	0.00	2.75
Salicylic acid	137.02	3.07	C7H6O3	Benzenoids	Benzene and substituted derivatives	C00805	0.00	2.15
Santene hydrate	139.11	4.59	C9H16O	Lipids and lipid‐like molecules	Prenol lipids	NA	0.03	1.11
Sayanedin	299.09	3.46	C17H14O5	Phenylpropanoids and polyketides	Isoflavonoids	C10527	0.02	2.10
Sciadonic acid	305.25	7.93	C20H34O2	Lipids and lipid‐like molecules	Fatty Acyls	NA	0.00	1.61
Sulfameter	281.07	3.56	C11H12N4O3S	NA	NA	NA	0.00	3.61
Tamoxifen	372.23	2.94	C26H29NO	Phenylpropanoids and polyketides	Stilbenes	C07108	0.00	2.30
Taurodeoxycholic acid	498.29	3.88	C26H45NO6S	Lipids and lipid‐like molecules	Sterol Lipids	C05463	0.02	1.90
Trachelanthine	302.19	3.17	C15H27NO5	Organoheterocyclic compounds	Indoles and derivatives	NA	0.00	1.61
Trilobinone	315.19	4.66	C20H28O3	Lipids and lipid‐like molecules	Prenol lipids	NA	0.01	1.40
Ursocholic acid	467.30	4.07	C24H40O5	Lipids and lipid‐like molecules	Sterol Lipids	C17644	0.00	2.54
Yucalexin A19	317.21	4.99	C20H30O3	Lipids and lipid‐like molecules	Prenol lipids	NA	0.02	1.64

### 3.6 Analysis of Serum Differential Metabolites in NX‐Treated OVX Rats

**FIGURE 8:**
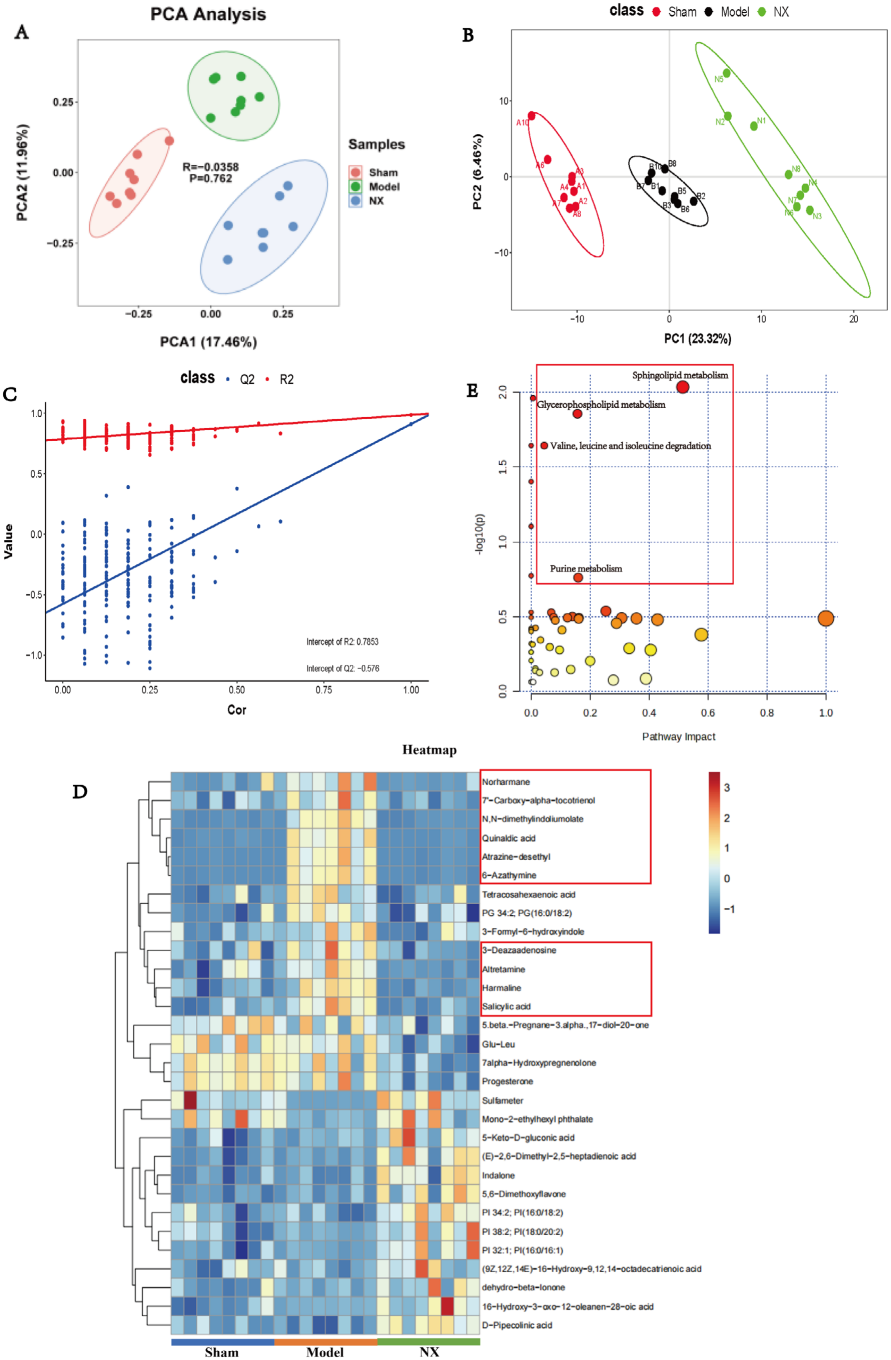
Metabolomic analysis of NX treatment, model and sham groups. (A) PCA analysis of NX; (B, C) PLS‐DA of NX; (D) heat map and cluster analysis of differential metabolites. (E) Functional enrichment analysis of NX differential metabolites.

The PCA and PLS‐DA results demonstrated significant within‐group clustering among the NX, sham, and model groups, indicating notable between‐group differences in the metabolites in these three groups (Figure 8A–C). The key potential biomarkers identified in the PLS‐DA analysis are presented in Table 4. Furthermore, the top 30 significant differential metabolites in the sham, model, and NX groups were presented in the clustered heatmap (Figure 8D). Among them, we found 10 differential metabolites that were up‐regulated following modelling while down‐regulated after NX treatment, namely, Norharmane, 7′‐carboxy‐alpha‐tocotrienol, N,N‐dimethylindoliumolate, quinaldic acid, atrazine‐desethyl, 6‐azathymine, 3‐deazaadenosine, altretamine, harmaline, and salicylic acid. These metabolites were primarily associated with lipid, amino acid, and sterol metabolism. Pathway impact analysis revealed that the most crucial metabolic pathways for the differential metabolites between the NX, sham, and model groups were sphingolipid, glycerophospholipid, valine, leucine, and isoleucine degradation, and purine metabolism (Figure 8E).

**TABLE 4:** All the differential metabolites screened among Sham, Model and NX groups (VIP > 1; *p* < 0.05).

Name	MZ	RT	Formula	SuperClass	Class	KEGG	*p*	VIP
D‐Pipecolinic acid	130.09	1.28	C6H11NO2	Organic acids and derivatives	Carboxylic acids and derivatives	NA	0.00	1.05
Oleoyl‐L‐carnitine	426.35	6.16	C25H47NO4	Lipids and lipid‐like molecules	Fatty Acyls	NA	0.02	1.07
Citronellyl acetate	216.19	3.90	C12H22O2	NA	NA	NA	0.00	1.65
Monoethylhexyl phthalic acid	277.14	4.18	C16H22O4	NA	NA	NA	0	3.29
Salicylic acid	137.02	3.07	C7H6O3	Benzenoids	Benzene and substituted derivatives	C00805	0.00	2.39
Catechol	109.03	2.96	C6H6O2	Benzenoids	Phenols	C00090	0.04	1.94
Palmitamide	256.26	7.33	C16H33NO	Lipids and lipid‐like molecules	Fatty Acyls	NA	0.01	1.13
Quinaldic acid	174.05	3.09	C10H7NO2	Organoheterocyclic compounds	Quinolines and derivatives	C06325	0	6.54
Fenchyl acetate	214.18	4.16	C12H20O2	Lipids and lipid‐like molecules	Fatty Acyls	NA	0.01	2.17
PI (18:3 (6Z,9Z,12Z)/22:3 (10Z,13Z,16Z))	909.55	7.74	C49H83O13P	Lipids and lipid‐like molecules	Glycerophospholipids	C01194	0.00	2.95
2,6‐Diisopropyl‐3‐methylphenol	193.16	4.38	C13H20O	Lipids and lipid‐like molecules	Prenol lipids	C12286	0.00	1.51
5‐Keto‐D‐gluconic acid	193.03	3.20	C6H10O7	Organic acids and derivatives	Hydroxy acids and derivatives	NA	0.01	1.44
PI (18:0/22:5 (4Z,7Z,10Z,13Z,16Z))	911.56	7.82	C49H85O13P	Lipids and lipid‐like molecules	Glycerophospholipids	C01194	0.00	1.68
Norharmane	169.07	3.10	C11H8N2	Organoheterocyclic compounds	Indoles and derivatives	C20157	0.00	3.70
PI (18:0/22:4 (10Z,13Z,16Z,19Z))	913.58	8.00	C49H87O13P	Lipids and lipid‐like molecules	Glycerophospholipids	C01194	0.00	3.00
2,6‐Dihydroxybenzoic acid	153.02	3.20	C7H6O4	Benzenoids	Benzene and substituted derivatives	NA	0.03	2.49
Heptanoylcarnitine	274.20	3.39	C14H28NO4	Lipids and lipid‐like molecules	Fatty Acyls	C02301	0.00	1.37
Pyrimidine	81.05	9.36	C4H4N2	Organoheterocyclic compounds	Diazines	C00396	0.02	1.60
L‐Alloisoleucine	130.09	1.62	NA	NA	NA	NA	0.00	1.11
PI (16:2 (9Z,12Z)/22:3 (10Z,13Z,16Z))	883.53	7.58	C47H81O13P	Lipids and lipid‐like molecules	Glycerophospholipids	C01194	0.00	1.29
Butyric acid	87.04	1.16	C4H8O2	Lipids and lipid‐like molecules	Fatty Acyls	C00246	0.00	1.18
3‐Methyl‐2‐oxovaleric acid	129.06	2.83	C6H10O3	Organic acids and derivatives	Keto acids and derivatives	C03465	0.00	1.26
PI 39:4; PI (19:0/20:4)	899.56	7.93	C48H85O13P	Lipids and lipid‐like molecules	Glycerophospholipids	C01194	0.00	2.44
Spermic acid 2	250.18	4.38	C15H23NO2	NA	NA	NA	0.00	1.61
PI (16:0/20:2 (11Z,14Z))	861.55	7.85	C45H83O13P	Lipids and lipid‐like molecules	Glycerophospholipids	C01194	0.00	1.93
3‐Indoleacrylic acid	186.06	3.77	C11H9NO2	Organoheterocyclic compounds	Indoles and derivatives	NA	0.04	1.49
Yucalexin A19	317.21	4.99	C20H30O3	Lipids and lipid‐like molecules	Prenol lipids	NA	0.00	2.76
PI (16:0/16:0)	809.51	7.76	C41H79O13P	Lipids and lipid‐like molecules	Glycerophospholipids	NA	0	4.58
Vanylglycol	183.07	3.08	C9H12O4	NA	NA	NA	0.00	2.00
Tauro‐b‐muricholic acid	514.28	3.67	C26H45NO7S	Lipids and lipid‐like molecules	Sterol Lipids	NA	0.01	2.12
p‐Cresol sulfate	187.01	3.31	C7H8O4S	Organic acids and derivatives	Organic sulfuric acids and derivatives	NA	0.04	1.12
2‐Piperidinone	100.08	2.85	C5H9NO	Organoheterocyclic compounds	Piperidines	NA	0.02	1.06
3‐Methylbenzaldehyde	119.05	3.45	C8H8O	Benzenoids	Benzene and substituted derivatives	C07209	0.01	1.03
Azaspiracid 2	871.53	7.69	C46H81O13P	Lipids and lipid‐like molecules	Glycerophospholipids	C01194	0.00	2.25
Etomidate	267.11	0.82	C11H14N4O4	Nucleosides, nucleotides, and analogue urine nucleosides		C01736	0.00	2.06
(2E)‐Decenoyl‐ACP	128.07	1.28	C6H11NO2	Organic acids and derivatives	Carboxylic acids and derivatives	C03969	0.00	1.48
PI 36:1; PI (18:0/18:1)	863.56	8.08	C45H85O13P	Lipids and lipid‐like molecules	Glycerophospholipids	C01194	0	3.65
PI (16:1 (9Z)/18:1 (11Z))	833.52	7.59	C43H79O13P	Lipids and lipid‐like molecules	Glycerophospholipids	C01194	0.00	1.47
Sulfametopyrazine	281.07	3.56	C11H12N4O3S	NA	NA	NA	0.00	5.51
PS (22:6 (4Z,7Z,10Z,13Z,16Z,19Z)/22:6 (4Z,7Z,109, 3.3,16Z,19.)	188.27	9	C48H81O13P	Lipids and lipid‐like molecules	Glycerophospholipids	C01194	0.00	2.56
Atrazine‐desethyl	188.07	4.12	C6H10ClN5	Organoheterocyclic compounds	Triazines	C06559	0	6.78
Pyrocatechol sulfate	188.99	2.96	C6H6O5S	Organic acids and derivatives	Organic sulfuric acids and derivatives	NA	0.03	2.29
PG (16:0/18:2 (9Z,12Z))	745.50	7.84	C40H75O10P	Lipids and lipid‐like molecules	Glycerophospholipids	C00344	0.01	1.97
alpha‐Ionene	175.15	4.38	C13H18	Benzenoids	Tetralins	NA	0.00	1.55
7′‐Carboxy‐alpha‐tocotrienol	345.21	4.56	C21H30O4	Organoheterocyclic compounds	Benzopyrans	NA	0.00	2.16
PI 39:5; PI (17:0/22:5)	897.55	7.70	C48H83O13P	Lipids and lipid‐like molecules	Glycerophospholipids	C01194	0.00	2.52
PI 40:7; PI (18:1/22:6)	907.53	7.52	C49H81O13P	Lipids and lipid‐like molecules	Glycerophospholipids	C01194	0.01	1.58
Mono‐2‐ethylhexyl phthalate	277.14	4.50	C16H22O4	Benzenoids	Benzene and substituted derivatives	C03343	0.02	1.05
Hexanoylglycine	172.10	3.02	C8H15NO3	Organic acids and derivatives	Carboxylic acids and derivatives	NA	0.00	1.67
5‐Hydroxytryptophol	176.07	3.46	C10H11NO2	Organoheterocyclic compounds	Indoles and derivatives	NA	0.00	6.32
PI (16:0/18:1 (9Z))	835.53	7.80	C43H81O13P	Lipids and lipid‐like molecules	Glycerophospholipids	C01194	0.00	1.72
dehydro‐beta‐Ionone	191.14	7.91	C13H18O	Lipids and lipid‐like molecules	Prenol lipids	NA	0.03	1.75
PS (18:2 (9Z,12Z)/22:6 (4Z,7Z,10Z,13Z,16Z,19Z))	847.53	7.72	C44H81O13P	Lipids and lipid‐like molecules	Glycerophospholipids	C01194		2.97
Taurocholate	516.30	3.85	C12H20O3	Lipids and lipid‐like molecules	Fatty Acyls	C16309	0.00	1.55
Traumatin	211.13	8.12	C47H87O13P	Lipids and lipid‐like molecules	Glycerophospholipids	C01194	0.00	2.71
PI (16:0/22:2 (13Z,16Z))	889.58	3.63	C21H32O4	Lipids and lipid‐like molecules	Sterol Lipids	C05475	0.01	1.85
3b,15b,17a‐Trihydroxy‐pregnenone	349.23	3.46	C8H8O4S	Organic acids and derivatives	Organic sulfuric acids and derivatives	NA	0.01	1.04
4‐Vinylphenol sulfate	199.01	4.66	C20H28O3	Lipids and lipid‐like molecules	Prenol lipids	NA	0.02	1.58
Trilobinone	315.19	3.58	C9H14O2	Lipids and lipid‐like molecules	Fatty Acyls	NA	0.00	1.21
(E)‐2,6‐Dimethyl‐2,5‐heptadienoic acid	153.09	3.10	C7H12O2	Organic acids and derivatives	Carboxylic acids and derivatives	NA	0.00	1.26
4‐Pentenyl acetate	127.08	3.93	C17H19N3	Organoheterocyclic compounds	Piperazinoazepines	C07570	0.00	1.21
Mirtazapine	266.16	7.75	C24H36O2	Lipids and lipid‐like molecules	Fatty Acyls	NA	0.00	1.43
Tetracosahexaenoic acid	355.26	3.42	C9H7NO2	Organoheterocyclic compounds	Indoles and derivatives	NA	0.02	1.69
3‐Formyl‐6‐hydroxyindole	160.04	5.11	C21H34O3	Lipids and lipid‐like molecules	Sterol Lipids	C14609	0.05	1.36
Dihydroceramide	352.28	3.95	C12H18O3	Lipids and lipid‐like molecules	Fatty Acyls	C08491	0.00	1.65
Jasmonic acid	209.12	2.92	C12H12N2O2	Organoheterocyclic compounds	Indoles and derivatives	NA	0	2.29
L‐1,2,3,4‐Tetrahydro‐beta‐carboline‐3‐carboxylic2a1c d.10	180.17	3.94	C12H21N	Organic nitrogen compounds	Organonitrogen compounds	C13736	0.00	2.11
Memantine	325.17	2.98	C20H21FN2O	Benzenoids	Benzene and substituted derivatives	C07572	0.02	2.32
5S,6S‐epoxy‐15R‐hydroxy‐ETE	293.21	5.63	C18H30O3	Lipids and lipid‐like molecules	Fatty Acyls	NA	0.03	1.07
Citalopram	807.50	4.02	C11H20N2O5	Organic acids and derivatives	Carboxylic acids and derivatives	NA	0.00	1.54
(9Z,12Z,14E)‐16‐Hydroxy‐9,12,14‐octadecatrieno26ic1a.i5d	859.52	7.54	C41H77O13P	Lipids and lipid‐like molecules	Glycerophospholipids	C01194	0.00	2.16
PI 36:3; PI(18:1/18:2)	464.30	7.55	C45H81O13P	Lipids and lipid‐like molecules	Glycerophospholipids	C01194	0.00	1.01
Glu‐Leu	175.03	2.84	C10H6O3	Phenylpropanoids and polyketides	Isocoumarins and derivatives	NA	0.05	1.11
PI (16:0/16:1 (9Z))	315.23	4.48	C21H30O2	Lipids and lipid‐like molecules	Sterol Lipids	C00410	0.01	1.83
PI 36:3; PI (18:1/18:2)	356.28	4.39	C20H38NO4	Lipids and lipid‐like molecules	Fatty Acyls	C02301	0.04	1.12
1‐Oxo‐1H‐2‐benzopyran‐3‐carboxaldehyde	266.15	4.38	C13H19N3O3	NA	NA	NA	0.00	1.64
Progesterone	210.11	3.08	C13H11N3	Organoheterocyclic compounds	Quinolines and derivatives	C11181	0.04	1.51
14,15‐DiHETrE	897.58	7.89	C49H86O12S	Lipids and lipid‐like molecules	Glycerolipids	C13508	0.01	1.08
Lysyl‐Proline	209.15	4.51	C9H18N6	Organic nitrogen compounds	Organonitrogen compounds	NA	0	3.94
Proflavine	109.10	3.52	C8H12	Hydrocarbons	Unsaturated hydrocarbons	NA	0.01	1.25
SQDG 40:4; SQDG (18:0/22:4)	215.12	3.24	C13H14N2O	Alkaloids and derivatives	Harmala alkaloids	C06536	0.00	2.40
Altretamine	469.33	5.83	C30H46O4	Lipids and lipid‐like molecules	Prenol lipids	NA	0.00	4.35
(Z)‐1,3‐Octadiene	128.03	3.19	C5H7NO3	Organic acids and derivatives	Carboxylic acids and derivatives	C01879	0.00	1.17
Harmaline	253.05	3.78	C15H10O4	Phenylpropanoids and polyketides	Flavonoids	C10028	0.03	4.00
16‐Hydroxy‐3‐oxo‐12‐oleanen‐28‐oic acid	256.22	4.91	C15H26O2	Lipids and lipid‐like molecules	Prenol lipids	NA	0.00	2.02
Pyroglutamic acid	821.51	7.67	C42H79O13P	Lipids and lipid‐like molecules	Glycerophospholipids	C01194	0.00	2.71
Chrysin	200.16	3.95	C11H18O2	Organic acids and derivatives	NA	NA	0.00	1.12
1,6,9‐Farnesatriene‐3,11‐diol	256.22	4.91	C15H26O2	Lipids and lipid‐like molecules	Prenol lipids	NA	0.00	1.11
Lactosylceramide (d18:1/12:0)	242.18	3.95	C9H10O3	NA	NA	NA	0	1.16
PS (16:1 (9Z)/22:6 (4Z,7Z,10Z,13Z,16Z,19Z))	184.09	0.76	C10H14ClN	NA	NA	NA	0.03	1.07
11‐nitro‐1‐undecene	161.13	4.15	C12H16	Lipids and lipid‐like molecules	Prenol lipids	NA	0.05	1.17
Malonyl‐Carnitin	242.18	3.95	C13H25NO3	Organic acids and derivatives	Carboxylic acids and derivatives	NA	0	2.21
1‐(1‐Methylethenyl)‐4‐(1‐methylethyl)benzene	843.50	7.40	C44H77O13P	Lipids and lipid‐like molecules	Glycerophospholipids	C01194	0.00	1.53
N‐Undecanoylglycine	269.13	3.56	C11H16N4O4	Organic acids and derivatives	Carboxylic acids and derivatives	NA	0.02	1.16
Azaspiracid 3	231.12	2.99	C11H13F3N2	Organoheterocyclic compounds	NA	NA	0.00	1.44
Dexrazoxane	830.49	7.42	C46H74NO10P	Lipids and lipid‐like molecules	Glycerophospholipids	C02737	0.00	1.82
Goyaglycoside h	175.05	2.60	C9H6N2O2	Organoheterocyclic compounds	Quinolines and derivatives	NA	0.03	1.12
N1‐Methyl‐2‐pyridone‐5‐carboxamide	211.17	4.38	C13H22O2	Lipids and lipid‐like molecules	Fatty Acyls	NA	0.00	1.56
Methyl (2E,6Z)‐dodecadienoate	283.09	3.57	C17H14O4	Phenylpropanoids and polyketides	Flavonoids	NA	0.02	1.07
5,6‐Dimethoxyflavone	187.04	0.80	C11H8O3	Benzenoids	Naphthalenes	NA	0.01	2.00
Isoplumbagin	186.11	3.10	C9H17NO3	Organic acids and derivatives	Carboxylic acids and derivatives	NA	0.01	1.50
N‐Heptanoylglycine	333.24	4.48	C21H32O3	Lipids and lipid‐like molecules	Sterol Lipids	C18038	0.00	1.74
7alpha‐Hydroxypregnenolone	819.50	7.44	C42H77O13P	Lipids and lipid‐like molecules	Glycerophospholipids	C01194	0.00	1.84
Tacrolimus	128.05	4.12	C4H5N3O2	NA	NA	NA	0	7.30
6‐Azathymine	436.16	3.22	C21H22O9	Phenylpropanoids and polyketides	Isoflavonoids	NA	0.00	1.61
Equol 7‐O‐glucuronide	228.19	4.38	C13H22O2	Lipids and lipid‐like molecules	Fatty Acyls	NA	0.00	1.59
Propyl 2,4‐decadienoate	277.14	3.92	C16H20O4	Benzenoids	Indanes	NA	0.00	2.35
Acetylpterosin C	299.09	3.46	C17H14O5	Phenylpropanoids and polyketides	Isoflavonoids	C10527	0.00	2.34
Sayanedin	227.13	3.59	C12H18O4	NA	NA	C19142	0.01	1.23

### 3.7 Potential Common Differential Metabolites and Differential Metabolic Pathways of PMOP in DG, FL and NX Treated OVX Rats

**FIGURE 9:**
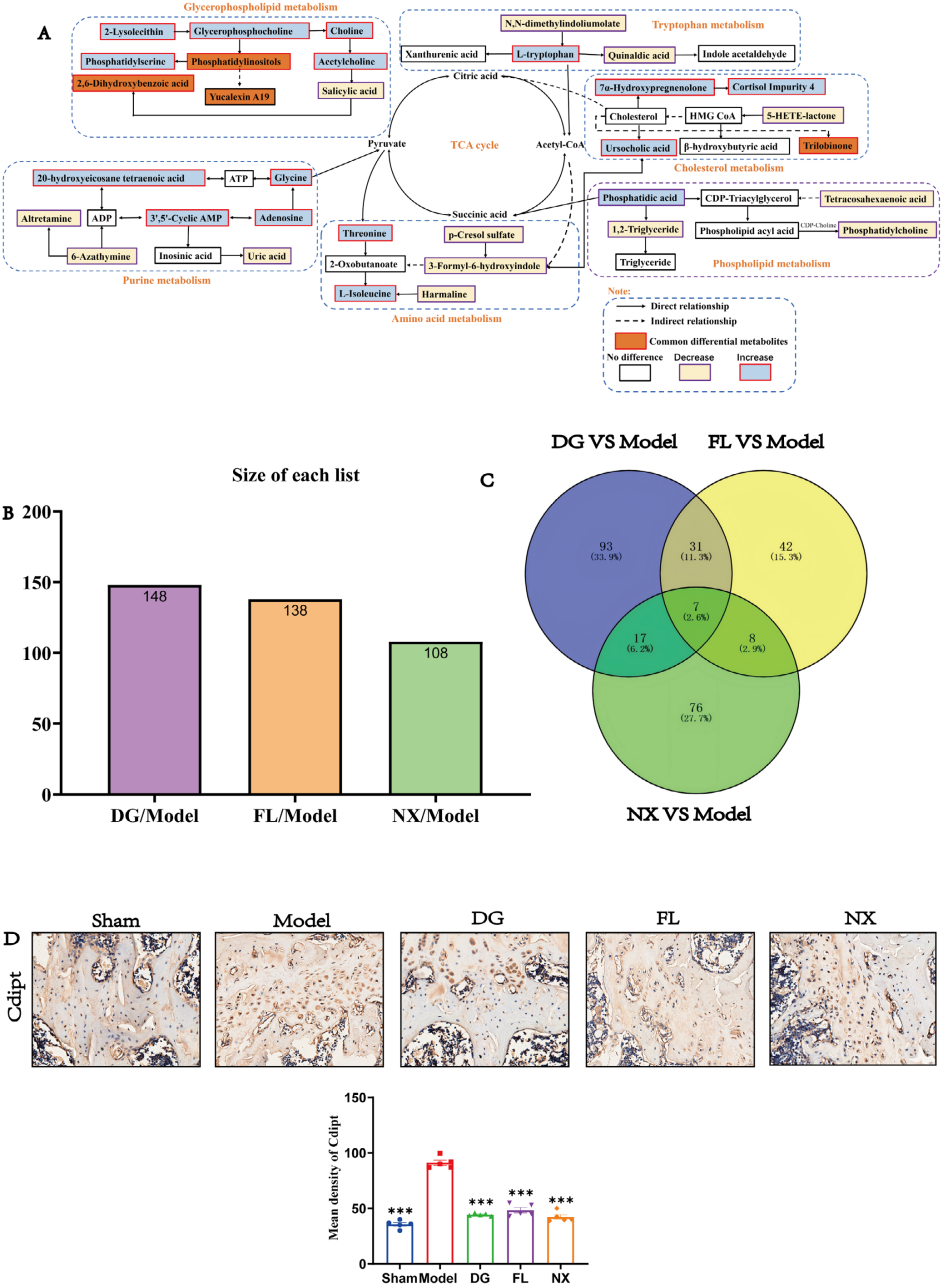
The common differential metabolites of DG, FL, and NX treatments were identified by Venn analysis (A‐B). (C) Metabolic networks of DG, FL, and NX on the basis of network analysis of hit targets and altered biomarkers to influence osteoporosis models. (D) Expression of phosphatidyl inositol synthase in OVX rats after DG, FL, and NX treatment. ***p < 0.001. Scale bar = 50 μm.

In this study, we separately compared the model group with the sham, DG, FL, and NX groups, to identify their differential metabolites (Table 5). Among them, there were 148 differential metabolites in DG, 138 differential metabolites in FL, and 108 differential metabolites in NX (Figure 9B). On the basis of the metabolic pathways identified in this study, we constructed perturbed metabolic networks which exhibited the relationship between the perturbed metabolic pathways, metabolites, and key target proteins. As shown in Figure 9A, DG, FL, and NX treatments were related to glycerophospholipid metabolism, phospholipid metabolism, tryptophan metabolism, purine metabolism, and amino acid metabolism. Furthermore, Venn analysis was performed to identify common differential metabolites, namely, 2,6‐Dihydroxybenzoic acid, PI 39:5; PI (17:0/22:5), PI 40:7; PI (18:1/22:6), Yucalexin A19, Trilobinone, PI 39:4; PI (19:0/20:4), PI 36:1; PI (18:0/18:1) (Figure 9C). We found that most of these are related to glycerophospholipid and sphingolipid metabolism (Figure 9A), so we speculate that DG, FL, and NX may all treat PMOP by affecting the lipid metabolism pathway.

**TABLE 5:** The metabolites of DG, FL and NX groups were different from those of Model group, and the yellow background was the common metabolites of the three drugs.

DG vs. model								
Name	MZ	RT	Formula	SuperClass	Class	KEGG	p	VIP
Lauroyl‐L‐carnitine	344.28	4.42	C19H37NO4	Lipids and lipid‐like molecules	Fatty Acyls	NA	0	1.2
Deoxycholic acid	391.28	5	C24H40O4	Lipids and lipid‐like molecules	NA	C04483	0.02	2.21
D‐Pipecolinic acid	130.09	1.28	C6H11NO2	Organic acids and derivatives	Carboxylic acids and derivatives	NA	0.01	1.04
Hexanoyl‐L‐carnitine	260.18	3.29	C13H25NO4	Lipids and lipid‐like molecules	Fatty Acyls	NA	0	1.19
O‐Acetyl‐L‐carnitine	204.12	1.2	C9H18NO4	Lipids and lipid‐like molecules	Fatty Acyls	C02571	0.01	1.03
Octanoylcarnitine	288.21	3.63	C15H29NO4	Lipids and lipid‐like molecules	Fatty Acyls	C02838	0	1.02
Cholic acid	407.28	4.36	C24H40O5	Lipids and lipid‐like molecules	Steroids and steroid derivatives	C00695	0	3.12
Oleoyl‐L‐carnitine	426.35	6.16	C25H47NO4	Lipids and lipid‐like molecules	Fatty Acyls	NA	0	2.21
Salicylic acid	137.02	3.07	C7H6O3	Benzenoids	Benzene and substituted derivatives	C00805	0	2.15
1‐O‐Hexadecyl‐2‐O‐butanoyl‐sn‐glyceryl‐3‐phosphocholine	552.4	7.39	C28H58NO7P	Lipids and lipid‐like molecules	Glycerophospholipids	NA	0	1.45
12R‐Hydroxy‐5Z,8Z,10E,14Z‐eicosatetraenoic acid	319.23	5.68	C20H32O3	Lipids and lipid‐like molecules	Fatty Acyls	NA	0	2.77
Quinaldic acid	174.05	3.09	C10H7NO2	Organoheterocyclic compounds	Quinolines and derivatives	C06325	0	7.35
3‐Hydroxyoleylcarnitine	442.35	5.15	C25H47NO5	Lipids and lipid‐like molecules	Fatty Acyls	NA	0	2.82
3,5‐Di‐tert‐butyl‐4‐hydroxybenzoic acid	251.16	3.7	C15H22O3	Benzenoids	Benzene and substituted derivatives	NA	0	1.03
Linoleoylcarnitine	424.34	5.55	C25H45NO4	Lipids and lipid‐like molecules	Fatty Acyls	NA	0.03	1.96
Acylcarnitine 16:1	398.32	5.27	C23H44NO4	Lipids and lipid‐like molecules	Fatty Acyls	C02301	0	1.93
Palmitoylcarnitine	400.34	5.88	C23H45NO4	Lipids and lipid‐like molecules	Fatty Acyls	C02990	0	1.8
1H‐Indole‐4‐carboxaldehyde	146.06	3.48	C9H7NO	Organoheterocyclic compounds	Indoles and derivatives	NA	0	1.59
Acylcarnitine 16:2	396.31	4.84	C23H42NO4	Lipids and lipid‐like molecules	Fatty Acyls	C02301	0	1.29
5‐Keto‐D‐gluconic acid	193.03	3.2	C6H10O7	Organic acids and derivatives	Hydroxy acids and derivatives	NA	0.01	1.31
Norharmane	169.07	3.1	C11H8N2	Organoheterocyclic compounds	Indoles and derivatives	C20157	0.05	1.42
Hippuric acid	178.05	2.94	C9H9NO3	Benzenoids	Benzene and substituted derivatives	C01586	0	2.67
PI 40:4; PI (18:0/22:4)	913.58	8	C49H87O13P	Lipids and lipid‐like molecules	Glycerophospholipids	C01194	0.01	1.92
LysoPC 18:2	578.34	5.2	C26H50NO7P	Lipids and lipid‐like molecules	Glycerophospholipids	C04230	0	1.13
2,6‐Dihydroxybenzoic acid	153.02	3.2	C7H6O4	Benzenoids	Benzene and substituted derivatives	NA	0	2.88
Acylcarnitine 14:0	372.31	5	C21H42NO4	Lipids and lipid‐like molecules	Fatty Acyls	C02301	0	1.89
Gamma‐Linolenic acid	277.22	6.88	C18H30O2	Lipids and lipid‐like molecules	Fatty Acyls	C06426	0.02	1.05
DL‐Indole‐3‐lactic acid	204.07	3.2	C11H11NO3	Organoheterocyclic compounds	Indoles and derivatives	NA	0	1.1
Pelargonic acid	157.12	4.69	C9H18O2	Lipids and lipid‐like molecules	Fatty Acyls	C01601	0.04	1.24
Octadecadienoate	279.23	7.47	C18H32O2	Lipids and lipid‐like molecules	Fatty Acyls	NA	0.01	1.06
Acylcarnitine 14:1	370.29	4.66	C21H40NO4	Lipids and lipid‐like molecules	Fatty Acyls	C02301	0	1.53
LysoPC 17:1	566.34	5.32	C25H50NO7P	Lipids and lipid‐like molecules	Glycerophospholipids	C04230	0	1.07
(R)‐3‐Hydroxybutyric acid	103.04	1.06	C4H8O3	Organic acids and derivatives	Hydroxy acids and derivatives	C01089	0	2.13
5‐Hydroxy‐6E,8Z,11Z,14Z‐eicosatetraenoic acid, 1,5‐lactone	303.23	5.68	C20H30O2	Lipids and lipid‐like molecules	Fatty Acyls	NA	0	2.53
Acylcarnitine 10:0	316.25	3.99	C17H34NO4	Lipids and lipid‐like molecules	Fatty Acyls	C02301	0	1.29
Sciadonic acid	305.25	7.93	C20H34O2	Lipids and lipid‐like molecules	Fatty Acyls	NA	0	1.61
Goshuyic acid	223.17	5.83	C14H24O2	Lipids and lipid‐like molecules	Fatty Acyls	NA	0.01	1.04
PI 38:5; PI (18:1/20:4)	883.53	7.58	C47H81O13P	Lipids and lipid‐like molecules	Glycerophospholipids	C01194	0	1.71
N‐Stearoyltaurine	390.27	5.54	C20H41NO4S	NA	NA	NA	0	1.1
LysoPC 20:3	604.36	5.48	C28H52NO7P	Lipids and lipid‐like molecules	Glycerophospholipids	C04230	0.01	2.69
Phenyl glucuronide	269.07	2.75	C12H14O7	NA	NA	NA	0.02	1.62
Acylcarnitine 8:1	286.2	3.49	C15H28NO4	Lipids and lipid‐like molecules	Fatty Acyls	C02301	0	1.2
PI 39:4; PI (19:0/20:4)	899.56	7.93	C48H85O13P	Lipids and lipid‐like molecules	Glycerophospholipids	C01194	0.03	1.59
Palmitelaidic acid	253.22	7.22	C16H30O2	Lipids and lipid‐like molecules	Fatty Acyls	NA	0	1.51
Phenol	93.03	3.05	C6H6O	Benzenoids	Phenols	C00146	0.02	1.22
Phenol sulphate	172.99	3.05	C6H6O4S	Organic acids and derivatives	Organic sulfuric acids and derivatives	C00850	0.01	1.55
PI 36:2; PI (18:0/18:2)	861.55	7.85	C45H83O13P	Lipids and lipid‐like molecules	Glycerophospholipids	C01194	0	1.19
Acylcarnitine 12:1	342.26	4.18	C19H36NO4	Lipids and lipid‐like molecules	Fatty Acyls	C02301	0.02	1.13
Taurodeoxycholic acid	498.29	3.88	C26H45NO6S	Lipids and lipid‐like molecules	Sterol Lipids	C05463	0.02	1.9
Yucalexin A19	317.21	4.99	C20H30O3	Lipids and lipid‐like molecules	Prenol lipids	NA	0.02	1.64
1,2‐Dipalmitoyl‐sn‐glycero‐3‐phospho‐(1′‐myo‐inositol)	809.51	7.76	C41H79O13P	Lipids and lipid‐like molecules	Glycerophospholipids	NA	0.02	1.35
3‐Furancarboxylic acid, tetrahydro‐4‐methylene‐5‐oxo‐2‐propyl‐, (2R,3S)‐rel—	183.07	3.08	C9H12O4	NA	NA	NA	0	2.22
FAHFA 36:3; FAHFA (18:2/18:1)	559.47	7.47	C36H64O4	Lipids and lipid‐like molecules	Fatty Acyls	NA	0.01	1.5
p‐Cresol sulfate	187.01	3.31	C7H8O4S	Organic acids and derivatives	Organic sulfuric acids and derivatives	NA	0	2.84
Oleic acid	281.25	8.18	C18H34O2	Lipids and lipid‐like molecules	Fatty Acyls	C00712	0	1.08
Acylcarnitine 4:0	232.14	2.82	C11H22NO4	Lipids and lipid‐like molecules	Fatty Acyls	C02301	0	1.7
(Z)‐9‐Heptadecenoic acid	267.23	7.7	C17H32O2	Lipids and lipid‐like molecules	Fatty Acyls	C16536	0	1.33
PC 38:4; PC (18:0/20:4)	868.6	9.63	C46H84NO8P	Lipids and lipid‐like molecules	Glycerophospholipids	C00157	0	1.1
Atractylenolide III	247.13	4.01	C15H20O3	Lipids and lipid‐like molecules	Prenol lipids	NA	0	1.32
PC 38:6; PC (16:0/22:6)	864.57	9.63	C46H80NO8P	Lipids and lipid‐like molecules	Glycerophospholipids	C00157	0.01	1.41
Hydrocinnamic acid	149.06	3.86	C9H10O2	Phenylpropanoids and polyketides	Phenylpropanoic acids	C05629	0	1.77
PI 37:4; PI (17:0/20:4)	871.53	7.69	C46H81O13P	Lipids and lipid‐like molecules	Glycerophospholipids	C01194	0.04	1.5
Kessyl glycol	253.18	4.31	C15H26O3	Organoheterocyclic compounds	Oxepanes	NA	0	2.02
(3beta, 8beta)‐3‐Hydroxy‐7(11)‐eremophilen‐12,8‐olide	249.15	4.81	C15H22O3	Lipids and lipid‐like molecules	Prenol lipids	NA	0	2.21
Acylcarnitine 9:1	300.21	3.67	C16H30NO4	Lipids and lipid‐like molecules	Fatty Acyls	C02301	0.04	1.06
(2E)‐Decenoyl‐ACP	128.07	1.28	C6H11NO2	Organic acids and derivatives	Carboxylic acids and derivatives	C03969	0	1.24
LysoPC 22:4	630.38	5.79	C30H54NO7P	Lipids and lipid‐like molecules	Glycerophospholipids	C04230	0	1.18
PI 36:1; PI (18:0/18:1)	863.56	8.08	C45H85O13P	Lipids and lipid‐like molecules	Glycerophospholipids	C01194	0	2.17
PI 34:2; PI (16:0/18:2)	833.52	7.59	C43H79O13P	Lipids and lipid‐like molecules	Glycerophospholipids	C01194	0.02	1.12
Plasmenyl‐PE 40:6; PE (P‐18:0/22:6)	774.54	8.49	C45H78NO7P	Lipids and lipid‐like molecules	Glycerophospholipids	C04756	0.04	1.48
Sulfameter	281.07	3.56	C11H12N4O3S	NA	NA	NA	0	3.61
PI 39:6; PI (17:0/22:6)	895.53	7.59	C48H81O13P	Lipids and lipid‐like molecules	Glycerophospholipids	C01194	0	2.3
Atrazine‐desethyl	188.07	4.12	C6H10ClN5	Organoheterocyclic compounds	Triazines	C06559	0	7.49
LysoPC 20:2	606.38	5.92	C28H54NO7P	Lipids and lipid‐like molecules	Glycerophospholipids	C04230	0	2
2‐Indolecarboxylic acid	162.05	3.41	C9H7NO2	Organoheterocyclic compounds	Indoles and derivatives	NA	0	2.14
Norharman	169.08	3.53	C11H8N2	Organoheterocyclic compounds	Indoles and derivatives	C20157	0	1.29
Plasmenyl‐PE 37:4; PE (P‐17:0/20:4)	736.53	8.47	C42H76NO7P	Lipids and lipid‐like molecules	Glycerophospholipids	C04756	0.02	3.2
7′‐Carboxy‐alpha‐tocotrienol	345.21	4.56	C21H30O4	Organoheterocyclic compounds	Benzopyrans	NA	0	3.45
PI 39:5; PI (17:0/22:5)	897.55	7.7	C48H83O13P	Lipids and lipid‐like molecules	Glycerophospholipids	C01194	0.01	2.03
PI 40:7; PI (18:1/22:6)	907.53	7.52	C49H81O13P	Lipids and lipid‐like molecules	Glycerophospholipids	C01194	0.01	1.65
Galanal A	317.21	5.3	C20H30O3	Organic oxygen compounds	Organooxygen compounds	NA	0	2.05
Ursocholic acid	467.3	4.07	C24H40O5	Lipids and lipid‐like molecules	Sterol Lipids	C17644	0	2.54
LysoPC 22:5	628.36	5.35	C30H52NO7P	Lipids and lipid‐like molecules	Glycerophospholipids	C04230	0	1
2‐Exo‐hydroxy‐1,8‐cineole	169.12	4.81	C10H18O2	Organoheterocyclic compounds	Oxanes	NA	0	1.12
N,N‐dimethylindoliumolate	176.07	3.46	C10H11NO2	Organoheterocyclic compounds	Indoles and derivatives	NA	0	6.08
Palmitoyl sphingomyelin	703.57	7.81	C39H79N2O6P	NA	NA	NA	0.01	1.32
all‐trans‐Retinoic acid	301.21	5.3	C20H28O2	Lipids and lipid‐like molecules	Prenol lipids	C00777	0	2.77
PI 35:2; PI (17:0/18:2)	847.53	7.72	C44H81O13P	Lipids and lipid‐like molecules	Glycerophospholipids	C01194	0	2.22
Retinyl ester	301.22	5.68	C20H30O2	Lipids and lipid‐like molecules	Prenol lipids	C02075	0	2.75
PI 38:2; PI (18:0/20:2)	889.58	8.12	C47H87O13P	Lipids and lipid‐like molecules	Glycerophospholipids	C01194	0	2.2
LysoPC 16:2	492.3	4.67	C24H46NO7P	Lipids and lipid‐like molecules	Glycerophospholipids	C04230	0	1.37
LysoPC 20:5	600.33	4.86	C28H48NO7P	Lipids and lipid‐like molecules	Glycerophospholipids	C04230	0	1.38
11.beta.,21‐Dihydroxy‐5.beta.‐pregnane‐3,20‐dione	349.23	3.63	C21H32O4	Lipids and lipid‐like molecules	Sterol Lipids	C05475	0	3.15
2,7,8‐Trimethyl‐2‐(.beta.‐carboxyethyl)‐6‐hydroxychroman	263.13	4.03	C15H20O4	NA	NA	NA	0	1.41
Trilobinone	315.19	4.66	C20H28O3	Lipids and lipid‐like molecules	Prenol lipids	NA	0.01	1.4
PC 34:2; PC (16:0/18:2)	816.57	9.63	C42H80NO8P	Lipids and lipid‐like molecules	Glycerophospholipids	C00157	0	1.96
.delta.‐Nonalactone	157.12	3.59	C9H16O2	Organoheterocyclic compounds	Lactones	NA	0.01	1.36
4‐Pentenyl acetate	127.08	3.1	C7H12O2	Organic acids and derivatives	Carboxylic acids and derivatives	NA	0	1.79
13′‐Carboxy‐alpha‐tocotrienol	453.3	5.82	C29H42O4	Lipids and lipid‐like molecules	Prenol lipids	NA	0.04	1.41
24‐Oxo‐1alpha,25‐dihydroxyvitamin D3	429.3	5.58	C27H42O4	Lipids and lipid‐like molecules	Sterol Lipids	NA	0	2.72
3‐Formyl‐6‐hydroxyindole	160.04	3.42	C9H7NO2	Organoheterocyclic compounds	Indoles and derivatives	NA	0.01	1.53
Isolongifolene, 4,5,9,10‐dehydro—	199.14	3.46	C15H20	Hydrocarbons	Polycyclic hydrocarbons	NA	0.03	1.53
3‐Oxo‐1,4,11 (13)‐eudesmatrien‐12‐oic acid	245.12	3.98	C15H18O3	Lipids and lipid‐like molecules	Prenol lipids	NA	0.01	1.17
15,16‐DiHODE	311.22	5.17	C18H32O4	Lipids and lipid‐like molecules	Fatty Acyls	NA	0	2.09
FAHFA 40:7; FAHFA (20:4/20:3)	607.47	7.31	C40H64O4	Lipids and lipid‐like molecules	Fatty Acyls	NA	0	1.17
Glu‐Leu	261.15	4.02	C11H20N2O5	Organic acids and derivatives	Carboxylic acids and derivatives	NA	0	1.63
3‐Isobutylglutaric acid	187.1	3.15	C9H16O4	NA	NA	NA	0.01	1.14
Acylcarnitine 13:1	356.28	4.39	C20H38NO4	Lipids and lipid‐like molecules	Fatty Acyls	C02301	0.02	1.3
2‐Butyl‐4‐methylphenol	163.11	4.39	C11H16O	Benzenoids	Benzene and substituted derivatives	NA	0	1.48
Isobutyl angelate	155.11	3.22	C9H16O2	Lipids and lipid‐like molecules	Fatty Acyls	NA	0.02	1.3
PC 36:4; PC (16:0/20:4)	840.57	5.68	C44H80NO8P	Lipids and lipid‐like molecules	Glycerophospholipids	C00157	0	2.16
Plasmenyl‐PE 40:5; PE (P‐18:0/22:5)	776.56	8.49	C45H80NO7P	Lipids and lipid‐like molecules	Glycerophospholipids	C04756	0.04	1.43
2‐Naphthaldehyde	155.05	1.02	C11H8O	Benzenoids	Naphthalenes	NA	0.01	1.51
Santene hydrate	139.11	4.59	C9H16O	Lipids and lipid‐like molecules	Prenol lipids	NA	0.03	1.11
18‐Hydroxy‐11‐dehydrotetrahydrocorticosterone	363.22	4.04	C21H32O5	Lipids and lipid‐like molecules	Sterol Lipids	NA	0	2.59
Acylcarnitine 13:0	358.29	4.57	C20H40NO4	Lipids and lipid‐like molecules	Fatty Acyls	C02301	0	1.29
D‐(+)‐Glucosamine	180.1	0.81	C6H13NO5	Organic oxygen compounds	Organooxygen compounds	C00329	0.05	3.7
2,3‐Diphenylpyrazine	233.11	4.06	C16H12N2	Organoheterocyclic compounds	Diazines	NA	0.02	1.08
PC (18:2 (9Z,12Z)/20:3 (5Z,8Z,11Z))	808.58	4.09	C46H82NO8P	Lipids and lipid‐like molecules	Glycerophospholipids	C00157	0	1.15
Diethyl suberate	231.16	3.63	C12H22O4	NA	NA	NA	0.03	1.2
Imiquimod	239.13	3.89	C14H16N4	Organoheterocyclic compounds	Quinolines and derivatives	NA	0	1.52
1,1′‐[1,12‐Dodecanediylbis(oxy)]bisbenzene	355.26	4.35	C24H34O2	Benzenoids	Phenol ethers	NA	0	3.39
1‐Hydroxyibuprofen	223.13	3.89	C13H18O3	Phenylpropanoids and polyketides	Phenylpropanoic acids	NA	0	1.32
Tamoxifen	372.23	2.94	C26H29NO	Phenylpropanoids and polyketides	Stilbenes	C07108	0	2.3
PC (16:0/16:1 (9Z))	732.55	9.65	C40H78NO8P	Lipids and lipid‐like molecules	Glycerophospholipids	C00157	0	1.72
1,2‐Benzenediol bis (trimethylsilyl) ether	253.11	3.78	C12H22O2Si2	Benzenoids	Benzene and substituted derivatives	NA	0	1.55
PI 35:4; PI (15:0/20:4)	843.5	7.4	C44H77O13P	Lipids and lipid‐like molecules	Glycerophospholipids	C01194	0	1.74
Trachelanthine	302.19	3.17	C15H27NO5	Organoheterocyclic compounds	Indoles and derivatives	NA	0	1.61
1,2‐Dilinoleoyl‐sn‐glycero‐3‐phosphocholine	782.56	7.81	C44H80NO8P	Lipids and lipid‐like molecules	Glycerophospholipids	NA	0.03	1.06
2,4‐Octadiene	111.12	3.62	C8H14	Hydrocarbons	Unsaturated hydrocarbons	NA	0.03	1.24
Hirsutine	369.22	3.83	C22H28N2O3	Alkaloids and derivatives	NA	NA	0	1.97
Prenyl caproate	185.15	4.59	C11H20O2	Lipids and lipid‐like molecules	Fatty Acyls	C13422	0.03	1.06
(1R)‐Chrysanthemolactone	186.16	3.62	C10H16O2	Organoheterocyclic compounds	Lactones	NA	0.03	1.17
Physoperuvine	200.13	3.58	C8H15NO	Alkaloids and derivatives	Tropane alkaloids	C10864	0.01	1.39
7alpha‐Hydroxypregnenolone	333.24	4.48	C21H32O3	Lipids and lipid‐like molecules	Sterol Lipids	C18038	0.05	1.55
N‐Desmethylmirtazapine	252.17	3.81	C16H17N3	NA	NA	NA	0.01	1.21
PI 33:2; PI (15:0/18:2)	819.5	7.44	C42H77O13P	Lipids and lipid‐like molecules	Glycerophospholipids	C01194	0	2.12
L‐3‐Phenyllactic acid	165.06	3.38	C9H10O3	Phenylpropanoids and polyketides	Phenylpropanoic acids	C05607	0.01	2.65
6‐Azathymine	128.05	4.12	C4H5N3O2	NA	NA	NA	0	7.92
5‐(2‐Furanyl)‐3,4‐dihydro‐2H‐pyrrole	134.06	2.93	C8H9NO	Organoheterocyclic compounds	Heteroaromatic compounds	NA	0	2.76
Equol 7‐O‐glucuronide	436.16	3.22	C21H22O9	Phenylpropanoids and polyketides	Isoflavonoids	NA	0	1.26
3′,5′‐Cyclic AMP	330.06	2.72	C10H12N5O6P	Nucleosides, nucleotides, and analogues	NA	NA	0	1.64
Dimethylhexa‐1,4‐diene	111.12	4.59	C8H14	Hydrocarbons	Unsaturated hydrocarbons	NA	0.03	1.05
cis‐3‐Octenyl propionate	185.15	3.63	C11H20O2	Lipids and lipid‐like molecules	Fatty Acyls	NA	0.03	1.17
3‐Methylazelaic acid	201.11	4.01	C10H18O4	Lipids and lipid‐like molecules	Fatty Acyls	NA	0.01	1.45
Sayanedin	299.09	3.46	C17H14O5	Phenylpropanoids and polyketides	Isoflavonoids	C10527	0.02	2.1
FAHFA 27:4; FAHFA (18:4/9:0)	431.31	5.34	C27H44O4	Lipids and lipid‐like molecules	Fatty Acyls	NA	0.01	3.77
Butralin	296.16	3.35	C14H21N3O4	NA	NA	C18582	0	1.7

FL vs. Model							
Name	MZ	RT	SuperClass	Class	KEGG	*p*	VIP
Hirsutine	369.22	3.83	Alkaloids and derivatives	NA	NA	0	1.4
Hippuric acid	178.05	2.94	Benzenoids	Benzene and substituted derivatives	C01586	0	1.71
2,6‐Dihydroxybenzoic acid	153.02	3.2	Benzenoids	Benzene and substituted derivatives	NA	0.01	1.34
3‐Methylbenzaldehyde	119.05	3.45	Benzenoids	Benzene and substituted derivatives	C07209	0	1.68
N‐Hydroxy‐1‐aminonaphthalene	160.07	2.78	Benzenoids	Naphthalenes	C14789	0.01	1.69
Mono‐2‐ethylhexyl phthalate	277.14	4.5	Benzenoids	Benzene and substituted derivatives	C03343	0	1.9
Ginkgolic acid I	345.24	5.8	Benzenoids	Benzene and substituted derivatives	C10794	0.03	1.83
Isoplumbagin	187.04	0.8	Benzenoids	Naphthalenes	NA	0	4.16
L‐Propionylcarnitine	218.14	1.81	Lipids and lipid‐like molecules	Fatty Acyls	C03017	0.01	1.24
1‐Heptadecanoyl‐sn‐glycero‐3‐phosphocholine	510.35	5.78	Lipids and lipid‐like molecules	Glycerophospholipids	NA	0.02	1.49
1‐O‐Hexadecyl‐2‐O‐butanoyl‐sn‐glyceryl‐3‐phosphocholine	552.4	7.39	Lipids and lipid‐like molecules	Glycerophospholipids	NA	0	1.45
LysoPS 18:0; LysoPS 18:0	319.23	5.68	Lipids and lipid‐like molecules	Fatty Acyls	NA	0.05	1.44
12R‐Hydroxy‐5Z,8Z,10E,14Z‐eicosatetraenoic acid	524.3	5.18	Lipids and lipid‐like molecules	Glycerophospholipids	C05974	0.02	1.96
PI 40:6; PI (18:0/22:6)	909.55	7.74	Lipids and lipid‐like molecules	Glycerophospholipids	C01194	0.02	1.48
PI 40:5; PI (18:0/22:5)	911.56	7.82	Lipids and lipid‐like molecules	Glycerophospholipids	C01194	0.01	1.17
PI 40:4; PI (18:0/22:4)	913.58	8	Lipids and lipid‐like molecules	Glycerophospholipids	C01194	0	2.35
LysoPC 14:0	526.31	4.79	Lipids and lipid‐like molecules	Glycerophospholipids	C04230	0	1.54
LysoPC 15:0	540.33	5.1	Lipids and lipid‐like molecules	Glycerophospholipids	C04230	0	1.06
LysoPC 17:1	566.34	5.32	Lipids and lipid‐like molecules	Glycerophospholipids	C04230	0	1.15
5‐Hydroxy‐6E,8Z,11Z,14Z‐eicosatetraenoic acid, 1,5‐lactone	303.23	5.68	Lipids and lipid‐like molecules	Fatty Acyls	NA	0.03	1.52
PI 36:4; PI (16:0/20:4)	857.52	7.55	Lipids and lipid‐like molecules	Glycerophospholipids	C01194	0	1.42
PI 40:8; PI (20:4/20:4)	905.51	7.31	Lipids and lipid‐like molecules	Glycerophospholipids	C01194	0	3.2
PI 38:5; PI (18:1/20:4)	883.53	7.58	Lipids and lipid‐like molecules	Glycerophospholipids	C01194	0	2.23
LysoPC 19:1	594.38	6.12	Lipids and lipid‐like molecules	Glycerophospholipids	C04230	0	1.15
LysoPC 20:3	604.36	5.48	Lipids and lipid‐like molecules	Glycerophospholipids	C04230	0.01	2.11
PI 39:4; PI (19:0/20:4)	899.56	7.93	Lipids and lipid‐like molecules	Glycerophospholipids	C01194	0	1.65
LysoPC 18:3	576.33	4.9	Lipids and lipid‐like molecules	Glycerophospholipids	C04230	0	1.05
Taurodeoxycholic acid	498.29	3.88	Lipids and lipid‐like molecules	Sterol Lipids	C05463	0	2.75
Yucalexin A19	317.21	4.99	Lipids and lipid‐like molecules	Prenol lipids	NA	0	2.53
1,2‐Dipalmitoyl‐sn‐glycero‐3‐phospho‐(1′‐myo‐inositol)	809.51	7.76	Lipids and lipid‐like molecules	Glycerophospholipids	NA	0	3.25
PI 38:6; PI (16:0/22:6)	881.51	7.47	Lipids and lipid‐like molecules	Glycerophospholipids	C01194	0	1.21
PC 38:6; PC (16:0/22:6)	864.57	9.63	Lipids and lipid‐like molecules	Glycerophospholipids	C00157	0	1.22
PI 37:4; PI (17:0/20:4)	871.53	7.69	Lipids and lipid‐like molecules	Glycerophospholipids	C01194	0	2.08
LysoPS 20:4; LysoPS 20:4	544.27	4.54	Lipids and lipid‐like molecules	Glycerophospholipids	C05974	0	1.58
PI 36:1; PI (18:0/18:1)	863.56	8.08	Lipids and lipid‐like molecules	Glycerophospholipids	C01194	0	2.04
PI 34:2; PI (16:0/18:2)	833.52	7.59	Lipids and lipid‐like molecules	Glycerophospholipids	C01194	0	1.68
PI 39:6; PI (17:0/22:6)	895.53	7.59	Lipids and lipid‐like molecules	Glycerophospholipids	C01194	0	2.58
LysoPC 20:2	606.38	5.92	Lipids and lipid‐like molecules	Glycerophospholipids	C04230	0	1.82
PI 39:5; PI (17:0/22:5)	897.55	7.7	Lipids and lipid‐like molecules	Glycerophospholipids	C01194	0	2.77
PI 40:7; PI (18:1/22:6)	907.53	7.52	Lipids and lipid‐like molecules	Glycerophospholipids	C01194	0	2.33
all‐trans‐Retinoic acid	301.21	5.3	Lipids and lipid‐like molecules	Prenol lipids	C00777	0.01	1.78
PI 34:1; PI (16:0/18:1)	835.53	7.8	Lipids and lipid‐like molecules	Glycerophospholipids	C01194	0	1.41
Oleamide	282.28	7.54	Lipids and lipid‐like molecules	Fatty Acyls	C19670	0	1.62
PI 35:2; PI (17:0/18:2)	847.53	7.72	Lipids and lipid‐like molecules	Glycerophospholipids	C01194	0	2.66
PI 38:2; PI (18:0/20:2)	889.58	8.12	Lipids and lipid‐like molecules	Glycerophospholipids	C01194	0	1.85
LysoPC 20:5	600.33	4.86	Lipids and lipid‐like molecules	Glycerophospholipids	C04230	0	1.27
Trilobinone	315.19	4.66	Lipids and lipid‐like molecules	Prenol lipids	NA	0	2.16
12 Hydroxy arachidonic acid	319.23	6.05	Lipids and lipid‐like molecules	Fatty Acyls	NA	0	2.6
24‐Oxo‐1alpha,25‐dihydroxyvitamin D3	429.3	5.58	Lipids and lipid‐like molecules	Sterol Lipids	NA	0	2.24
PI 32:1; PI (16:0/16:1)	807.5	7.54	Lipids and lipid‐like molecules	Glycerophospholipids	C01194	0	2.33
PI 36:3; PI (18:1/18:2)	859.52	7.55	Lipids and lipid‐like molecules	Glycerophospholipids	C01194	0	1.22
SQDG 40:4; SQDG (18:0/22:4)	897.58	7.89	Lipids and lipid‐like molecules	Glycerolipids	C13508	0.03	2.19
12S‐HHT	279.2	4.9	Lipids and lipid‐like molecules	Fatty Acyls	NA	0	2.45
PI 33:1; PI (18:0/15:1)	821.51	7.67	Lipids and lipid‐like molecules	Glycerophospholipids	C01194	0	2.94
PI 35:4; PI (15:0/20:4)	843.5	7.4	Lipids and lipid‐like molecules	Glycerophospholipids	C01194	0	2.22
PI 33:2; PI (15:0/18:2)	819.5	7.44	Lipids and lipid‐like molecules	Glycerophospholipids	C01194	0	2.59
SQDG 38:3; SQDG (18:0/20:3)	871.57	7.92	Lipids and lipid‐like molecules	Glycerolipids	C13508	0.03	2.18
ent‐16b,19‐Kauranediol 19‐acetate	347.26	6.22	Lipids and lipid‐like molecules	Prenol lipids	NA	0.05	1.61
FAHFA 27:4; FAHFA (18:4/9:0)	431.31	5.34	Lipids and lipid‐like molecules	Fatty Acyls	NA	0	3.2
Sulfameter	281.07	3.56	NA	NA	NA	0	5.14
N‐Docosanoyltaurine	446.33	6.76	NA	NA	NA	0.01	1.41
Dodemorph	282.28	6.31	NA	NA	C18786	0.02	1.58
9‐Oxo‐11‐(3‐pentyl‐2‐oxiranyl)‐10E‐undecenoic acid	309.21	4.88	NA	NA	NA	0	1.03
Inosine 5’‐monophosphate	349.05	1.32	Nucleosides, nucleotides, and analogues	Purine nucleotides	C00130	0.04	2.08
Adenosine 3′‐monophosphate	346.05	1.66	Nucleosides, nucleotides, and analogues	Ribonucleoside 3′‐phosphates	C01367	0	2.17
3′,5′‐Cyclic AMP	330.06	2.72	Nucleosides, nucleotides, and analogues	NA	NA	0.04	1.38
Taurine	124.01	0.71	Organic acids and derivatives	Organic sulfonic acids and derivatives	C00245	0	1.03
Proline betaine	144.1	0.8	Organic acids and derivatives	Carboxylic acids and derivatives	C10172	0	1.69
(S)‐Homostachydrine	158.12	1.34	Organic acids and derivatives	Carboxylic acids and derivatives	C08283	0.01	1.41
N‐Acetyl‐D‐norleucine	172.1	3.02	Organic acids and derivatives	Carboxylic acids and derivatives	NA	0	1.21
4‐Vinylphenol sulfate	199.01	3.46	Organic acids and derivatives	Organic sulfuric acids and derivatives	NA	0	1.75
Trimethylamine N‐oxide	76.08	0.8	Organic nitrogen compounds	Organonitrogen compounds	C01104	0	2.94
D‐erythro‐Sphinganine	302.3	9.65	Organic nitrogen compounds	Organonitrogen compounds	C00836	0.01	1.22
Glyceraldehyde	89.03	0.79	Organic oxygen compounds	Organooxygen compounds	C02154	0	1.22
Galanal A	317.21	5.3	Organic oxygen compounds	Organooxygen compounds	NA	0.01	1.63
3‐Mercapto‐2‐butanone	105.04	3.42	Organic oxygen compounds	Organooxygen compounds	NA	0.01	1.13
2(3H)‐Benzothiazolethione	167.99	4.19	Organoheterocyclic compounds	Benzothiazoles	C14437	0.04	1.05
Uric acid	167.02	0.79	Organoheterocyclic compounds	Imidazopyrimidines	C00366	0.04	1.23
Serotonin	177.1	2.77	Organoheterocyclic compounds	Indoles and derivatives	C00780	0.01	1.68
N‐Acetylserotonin	219.11	3.23	Organoheterocyclic compounds	Indoles and derivatives	C00978	0	2.58
2‐Indolecarboxylic acid	162.05	3.41	Organoheterocyclic compounds	Indoles and derivatives	NA	0	1.11
8‐Hydroxyquinoline‐2‐carbaldehyde	191.08	2.79	Organoheterocyclic compounds	Quinolines and derivatives	NA	0.01	1.68
3‐Indoleacetic acid	174.06	3.61	Organoheterocyclic compounds	Indoles and derivatives	C00954	0	1.09
5‐(2‐Furanyl)‐3,4‐dihydro‐2H‐pyrrole	134.06	2.93	Organoheterocyclic compounds	Heteroaromatic compounds	NA	0	1.72
Hydrocinnamic acid	149.06	3.86	Phenylpropanoids and polyketides	Phenylpropanoic acids	C05629	0	1.53
1‐Oxo‐1H‐2‐benzopyran‐3‐carboxaldehyde	175.03	2.84	Phenylpropanoids and polyketides	Isocoumarins and derivatives	NA	0	1.09
Chrysin	253.05	3.78	Phenylpropanoids and polyketides	Flavonoids	C10028	0.05	1.36
L‐3‐Phenyllactic acid	165.06	3.38	Phenylpropanoids and polyketides	Phenylpropanoic acids	C05607	0.04	1.2

NX vs. Model								
Name	MZ	RT	Formula	SuperClass	Class	KEGG	*p*	VIP
D‐Pipecolinic acid	130.09	1.28	C6H11NO2	Organic acids and derivatives	Carboxylic acids and derivatives	NA	0	1.05
Oleoyl‐L‐carnitine	426.35	6.16	C25H47NO4	Lipids and lipid‐like molecules	Fatty Acyls	NA	0.02	1.07
Citronellyl acetate	216.19	3.9	C12H22O2	NA	NA	NA	0	1.65
Monoethylhexyl phthalic acid	277.14	4.18	C16H22O4	NA	NA	NA	0	3.29
Salicylic acid	137.02	3.07	C7H6O3	Benzenoids	Benzene and substituted derivatives	C00805	0	2.39
Catechol	109.03	2.96	C6H6O2	Benzenoids	Phenols	C00090	0.04	1.94
Palmitamide	256.26	7.33	C16H33NO	Lipids and lipid‐like molecules	Fatty Acyls	NA	0.01	1.13
Quinaldic acid	174.05	3.09	C10H7NO2	Organoheterocyclic compounds	Quinolines and derivatives	C06325	0	6.54
Fenchyl acetate	214.18	4.16	C12H20O2	Lipids and lipid‐like molecules	Fatty Acyls	NA	0.01	2.17
PI (18:3 (6Z,9Z,12Z)/22:3 (10Z,13Z,16Z))	909.55	7.74	C49H83O13P	Lipids and lipid‐like molecules	Glycerophospholipids	C01194	0	2.95
2,6‐Diisopropyl‐3‐methylphenol	193.16	4.38	C13H20O	Lipids and lipid‐like molecules	Prenol lipids	C12286	0	1.51
5‐Keto‐D‐gluconic acid	193.03	3.2	C6H10O7	Organic acids and derivatives	Hydroxy acids and derivatives	NA	0.01	1.44
PI (18:0/22:5 (4Z,7Z,10Z,13Z,16Z))	911.56	7.82	C49H85O13P	Lipids and lipid‐like molecules	Glycerophospholipids	C01194	0	1.68
Norharmane	169.07	3.1	C11H8N2	Organoheterocyclic compounds	Indoles and derivatives	C20157	0	3.7
PI (18:0/22:4 (10Z,13Z,16Z,19Z))	913.58	8	C49H87O13P	Lipids and lipid‐like molecules	Glycerophospholipids	C01194	0	3
2,6‐Dihydroxybenzoic acid	153.02	3.2	C7H6O4	Benzenoids	Benzene and substituted derivatives	NA	0.03	2.49
Heptanoylcarnitine	274.2	3.39	C14H28NO4	Lipids and lipid‐like molecules	Fatty Acyls	C02301	0	1.37
Pyrimidine	81.05	9.36	C4H4N2	Organoheterocyclic compounds	Diazines	C00396	0.02	1.6
L‐Alloisoleucine	130.09	1.62	NA	NA	NA	NA	0	1.11
PI (16:2 (9Z,12Z)/22:3 (10Z,13Z,16Z))	883.53	7.58	C47H81O13P	Lipids and lipid‐like molecules	Glycerophospholipids	C01194	0	1.29
Butyric acid	87.04	1.16	C4H8O2	Lipids and lipid‐like molecules	Fatty Acyls	C00246	0	1.18
3‐Methyl‐2‐oxovaleric acid	129.06	2.83	C6H10O3	Organic acids and derivatives	Keto acids and derivatives	C03465	0	1.26
PI 39:4; PI (19:0/20:4)	899.56	7.93	C48H85O13P	Lipids and lipid‐like molecules	Glycerophospholipids	C01194	0	2.44
Spermic acid 2	250.18	4.38	C15H23NO2	NA	NA	NA	0	1.61
PI (16:0/20:2 (11Z,14Z))	861.55	7.85	C45H83O13P	Lipids and lipid‐like molecules	Glycerophospholipids	C01194	0	1.93
3‐Indoleacrylic acid	186.06	3.77	C11H9NO2	Organoheterocyclic compounds	Indoles and derivatives	NA	0.04	1.49
Yucalexin A19	317.21	4.99	C20H30O3	Lipids and lipid‐like molecules	Prenol lipids	NA	0	2.76
PI (16:0/16:0)	809.51	7.76	C41H79O13P	Lipids and lipid‐like molecules	Glycerophospholipids	NA	0	4.58
Vanylglycol	183.07	3.08	C9H12O4	NA	NA	NA	0	2
Tauro‐b‐muricholic acid	514.28	3.67	C26H45NO7S	Lipids and lipid‐like molecules	Sterol Lipids	NA	0.01	2.12
p‐Cresol sulfate	187.01	3.31	C7H8O4S	Organic acids and derivatives	Organic sulfuric acids and derivatives	NA	0.04	1.12
2‐Piperidinone	100.08	2.85	C5H9NO	Organoheterocyclic compounds	Piperidines	NA	0.02	1.06
3‐Methylbenzaldehyde	119.05	3.45	C8H8O	Benzenoids	Benzene and substituted derivatives	C07209	0.01	1.03
Azaspiracid 2	871.53	7.69	C46H81O13P	Lipids and lipid‐like molecules	Glycerophospholipids	C01194	0	2.25
Etomidate	267.11	0.82	C11H14N4O4	Nucleosides, nucleotides, and analogues	Purine nucleosides	C01736	0	2.06
(2E)‐Decenoyl‐ACP	128.07	1.28	C6H11NO2	Organic acids and derivatives	Carboxylic acids and derivatives	C03969	0	1.48
PI 36:1; PI (18:0/18:1)	863.56	8.08	C45H85O13P	Lipids and lipid‐like molecules	Glycerophospholipids	C01194	0	3.65
PI (16:1 (9Z)/18:1 (11Z))	833.52	7.59	C43H79O13P	Lipids and lipid‐like molecules	Glycerophospholipids	C01194	0	1.47
Sulfametopyrazine	281.07	3.56	C11H12N4O3S	NA	NA	NA	0	5.51
PS (22:6 (4Z,7Z,10Z,13Z,16Z,19Z)/22:6 (4Z,7Z,10Z,13Z,16Z,19Z))	895.53	7.59	C48H81O13P	Lipids and lipid‐like molecules	Glycerophospholipids	C01194	0	2.56
	188.07	4.12	C6H10ClN5	Organoheterocyclic compounds	Triazines	C06559	0	6.78
Atrazine‐desethyl	188.99	2.96	C6H6O5S	Organic acids and derivatives	Organic sulfuric acids and derivatives	NA	0.03	2.29
Pyrocatechol sulfate	745.5	7.84	C40H75O10P	Lipids and lipid‐like molecules	Glycerophospholipids	C00344	0.01	1.97
PG (16:0/18:2 (9Z,12Z))	175.15	4.38	C13H18	Benzenoids	Tetralins	NA	0	1.55
alpha‐Ionene	345.21	4.56	C21H30O4	Organoheterocyclic compounds	Benzopyrans	NA	0	2.16
7′‐Carboxy‐alpha‐tocotrienol	897.55	7.7	C48H83O13P	Lipids and lipid‐like molecules	Glycerophospholipids	C01194	0	2.52
PI 39:5; PI (17:0/22:5)	907.53	7.52	C49H81O13P	Lipids and lipid‐like molecules	Glycerophospholipids	C01194	0.01	1.58
PI 40:7; PI (18:1/22:6)	277.14	4.5	C16H22O4	Benzenoids	Benzene and substituted derivatives	C03343	0.02	1.05
Mono‐2‐ethylhexyl phthalate	172.1	3.02	C8H15NO3	Organic acids and derivatives	Carboxylic acids and derivatives	NA	0	1.67
Hexanoylglycine	176.07	3.46	C10H11NO2	Organoheterocyclic compounds	Indoles and derivatives	NA	0	6.32
5‐Hydroxytryptophol	835.53	7.8	C43H81O13P	Lipids and lipid‐like molecules	Glycerophospholipids	C01194	0	1.72
PI (16:0/18:1 (9Z))	191.14	7.91	C13H18O	Lipids and lipid‐like molecules	Prenol lipids	NA	0.03	1.75
dehydro‐beta‐Ionone	847.53	7.72	C44H81O13P	Lipids and lipid‐like molecules	Glycerophospholipids	C01194	0	2.97
PS (18:2 (9Z,12Z)/22:6 (4Z,7Z,10Z,13Z,16Z,19Z))	211.13	3.85	C12H20O3	Lipids and lipid‐like molecules	Fatty Acyls	C16309	0	1.55
Traumatin	889.58	8.12	C47H87O13P	Lipids and lipid‐like molecules	Glycerophospholipids	C01194	0	2.71
PI (16:0/22:2 (13Z,16Z))	349.23	3.63	C21H32O4	Lipids and lipid‐like molecules	Sterol Lipids	C05475	0.01	1.85
3b,15b,17a‐Trihydroxy‐pregnenone	199.01	3.46	C8H8O4S	Organic acids and derivatives	Organic sulfuric acids and derivatives	NA	0.01	1.04
4‐Vinylphenol sulfate	315.19	4.66	C20H28O3	Lipids and lipid‐like molecules	Prenol lipids	NA	0.02	1.58
Trilobinone	153.09	3.58	C9H14O2	Lipids and lipid‐like molecules	Fatty Acyls	NA	0	1.21
(E)‐2,6‐Dimethyl‐2,5‐heptadienoic acid	127.08	3.1	C7H12O2	Organic acids and derivatives	Carboxylic acids and derivatives	NA	0	1.26
4‐Pentenyl acetate	266.16	3.93	C17H19N3	Organoheterocyclic compounds	Piperazinoazepines	C07570	0	1.21
Mirtazapine	355.26	7.75	C24H36O2	Lipids and lipid‐like molecules	Fatty Acyls	NA	0	1.43
Tetracosahexaenoic acid	160.04	3.42	C9H7NO2	Organoheterocyclic compounds	Indoles and derivatives	NA	0.02	1.69
3‐Formyl‐6‐hydroxyindole	352.28	5.11	C21H34O3	Lipids and lipid‐like molecules	Sterol Lipids	C14609	0.05	1.36
Dihydroceramide	209.12	3.95	C12H18O3	Lipids and lipid‐like molecules	Fatty Acyls	C08491	0	1.65
Jasmonic acid	217.1	2.92	C12H12N2O2	Organoheterocyclic compounds	Indoles and derivatives	NA	0	2.29
L‐1,2,3,4‐Tetrahydro‐beta‐carboline‐3‐carboxylic acid	180.17	3.94	C12H21N	Organic nitrogen compounds	Organonitrogen compounds	C13736	0	2.11
Memantine	209.14	2.99	C9H20O5	NA	NA	NA	0	1.18
5S,6S‐epoxy‐15R‐hydroxy‐ETE	325.17	2.98	C20H21FN2O	Benzenoids	Benzene and substituted derivatives	C07572	0.02	2.32
Citalopram	293.21	5.63	C18H30O3	Lipids and lipid‐like molecules	Fatty Acyls	NA	0.03	1.07
(9Z,12Z,14E)‐16‐Hydroxy‐9,12,14‐octadecatrienoic acid	261.15	4.02	C11H20N2O5	Organic acids and derivatives	Carboxylic acids and derivatives	NA	0	1.54
Glu‐Leu	807.5	7.54	C41H77O13P	Lipids and lipid‐like molecules	Glycerophospholipids	C01194	0	2.16
PI (16:0/16:1 (9Z))	859.52	7.55	C45H81O13P	Lipids and lipid‐like molecules	Glycerophospholipids	C01194	0	1.01
PI 36:3; PI (18:1/18:2)	175.03	2.84	C10H6O3	Phenylpropanoids and polyketides	Isocoumarins and derivatives	NA	0.05	1.11
1‐Oxo‐1H‐2‐benzopyran‐3‐carboxaldehyde	315.23	4.48	C21H30O2	Lipids and lipid‐like molecules	Sterol Lipids	C00410	0.01	1.83
Progesterone	356.28	4.39	C20H38NO4	Lipids and lipid‐like molecules	Fatty Acyls	C02301	0.04	1.12
14,15‐DiHETrE	266.15	4.38	C13H19N3O3	NA	NA	NA	0	1.64
Lysyl‐Proline	210.11	3.08	C13H11N3	Organoheterocyclic compounds	Quinolines and derivatives	C11181	0.04	1.51
Proflavine	897.58	7.89	C49H86O12S	Lipids and lipid‐like molecules	Glycerolipids	C13508	0.01	1.08
SQDG 40:4; SQDG (18:0/22:4)	209.15	4.51	C9H18N6	Organic nitrogen compounds	Organonitrogen compounds	NA	0	3.94
Altretamine	109.1	3.52	C8H12	Hydrocarbons	Unsaturated hydrocarbons	NA	0.01	1.25
(Z)‐1,3‐Octadiene	215.12	3.24	C13H14N2O	Alkaloids and derivatives	Harmala alkaloids	C06536	0	2.4
Harmaline	469.33	5.83	C30H46O4	Lipids and lipid‐like molecules	Prenol lipids	NA	0	4.35
16‐Hydroxy‐3‐oxo‐12‐oleanen‐28‐oic acid	128.03	3.19	C5H7NO3	Organic acids and derivatives	Carboxylic acids and derivatives	C01879	0	1.17
Pyroglutamic acid	253.05	3.78	C15H10O4	Phenylpropanoids and polyketides	Flavonoids	C10028	0.03	4
Chrysin	256.22	4.91	C15H26O2	Lipids and lipid‐like molecules	Prenol lipids	NA	0	2.02
1,6,9‐Farnesatriene‐3,11‐diol	806.56	9.65	C46H80NO8P	Lipids and lipid‐like molecules	Glycerophospholipids	C00157	0.04	1.16
Lactosylceramide (d18:1/12:0)	821.51	7.67	C42H79O13P	Lipids and lipid‐like molecules	Glycerophospholipids	C01194	0	2.71
PS (16:1 (9Z)/22:6 (4Z,7Z,10Z,13Z,16Z,19Z))	200.16	3.95	C11H18O2	Organic acids and derivatives	NA	NA	0	1.12
11‐nitro‐1‐undecene	184.09	0.76	C10H14ClN	NA	NA	NA	0.03	1.07
Malonyl‐Carnitin	161.13	4.15	C12H16	Lipids and lipid‐like molecules	Prenol lipids	NA	0.05	1.17
1‐(1‐Methylethenyl)‐4‐(1‐methylethyl)benzene	242.18	3.95	C13H25NO3	Organic acids and derivatives	Carboxylic acids and derivatives	NA	0	2.21
N‐Undecanoylglycine	843.5	7.4	C44H77O13P	Lipids and lipid‐like molecules	Glycerophospholipids	C01194	0	1.53
Azaspiracid 3	269.13	3.56	C11H16N4O4	Organic acids and derivatives	Carboxylic acids and derivatives	NA	0.02	1.16
Dexrazoxane	830.49	7.42	C46H74NO10P	Lipids and lipid‐like molecules	Glycerophospholipids	C02737	0	1.82
Goyaglycoside h	175.05	2.6	C9H6N2O2	Organoheterocyclic compounds	Quinolines and derivatives	NA	0.03	1.12
N1‐Methyl‐2‐pyridone‐5‐carboxamide	211.17	4.38	C13H22O2	Lipids and lipid‐like molecules	Fatty Acyls	NA	0	1.56
Methyl (2E,6Z)‐dodecadienoate	283.09	3.57	C17H14O4	Phenylpropanoids and polyketides	Flavonoids	NA	0.02	1.07
5,6‐Dimethoxyflavone	187.04	0.8	C11H8O3	Benzenoids	Naphthalenes	NA	0.01	2
Isoplumbagin	186.11	3.1	C9H17NO3	Organic acids and derivatives	Carboxylic acids and derivatives	NA	0.01	1.5
N‐Heptanoylglycine	333.24	4.48	C21H32O3	Lipids and lipid‐like molecules	Sterol Lipids	C18038	0	1.74
7alpha‐Hydroxypregnenolone	819.5	7.44	C42H77O13P	Lipids and lipid‐like molecules	Glycerophospholipids	C01194	0	1.84
Tacrolimus	128.05	4.12	C4H5N3O2	NA	NA	NA	0	7.3
6‐Azathymine	436.16	3.22	C21H22O9	Phenylpropanoids and polyketides	Isoflavonoids	NA	0	1.61
Equol 7‐O‐glucuronide	228.19	4.38	C13H22O2	Lipids and lipid‐like molecules	Fatty Acyls	NA	0	1.59
Propyl 2,4‐decadienoate	277.14	3.92	C16H20O4	Benzenoids	Indanes	NA	0	2.35
Acetylpterosin C	299.09	3.46	C17H14O5	Phenylpropanoids and polyketides	Isoflavonoids	C10527	0	2.34
Sayanedin	227.13	3.59	C12H18O4	NA	NA	C19142	0.01	1.23
3,4‐Methylenesebacic acid								

To verify the regulation of these three herbs on glycerophospholipid metabolism, we performed immunohistochemical staining to evaluate the expression level of Cdipt (phosphatidylinositol synthetase), a rate‐limiting enzyme in glycerophospholipid biosynthesis. The result showed that the Cdipt expression in the model group was up‐regulated compared with the sham group, while after DG, FL, and NX treatments, its expression was significantly down‐regulated (Figure 9D).

## 4 Discussion

In this study, our serum metabolomics analysis showed that DG mainly affected pregnenolone lipid metabolism, steroid lipid metabolism, and glycerophospholipid metabolism. Several key active ingredients in DG have been reported to regulate glycerophospholipid and cholesterol metabolism as well as lipid deposition [37, 38, 39]. In addition, we found that FL mainly affected glycerophospholipid metabolism, tryptophan, valine, leucine, and isoleucine degradation. In high‐fat diet‐fed mice, FL could significantly suppress lipid metabolism disorders by controlling the metabolisms of glycerophospholipids, unsaturated fatty acids, amino acids, choline, bile acids, tryptophan, and sphingolipids [40]. Furthermore, we detected that NX mainly affected sphingolipid metabolism, valine, leucine, isoleucine degradation, glycerophospholipid metabolism, and purine metabolism. Other studies also reported that the osteoprotective effect of the polysaccharide components of NX was related to the regulation of amino acid metabolism [41]. After 8 weeks of NX treatment in type 2 diabetes models, total cholesterol, triglycerides, low‐density lipoprotein cholesterol, and high‐density lipoprotein cholesterol levels in blood were improved significantly [42]. In summary, our data suggest that DG, FL, and NX are all related to lipid metabolism, which plays a pivotal role in the pathogenesis of osteoporosis [43].

Abnormal lipid metabolism can lead to impaired vascular endothelial function, induce atherosclerosis, and affect blood circulation [36, 44]. Thus, maintaining lipid metabolism homeostasis can invigorate blood, improve bone marrow microcirculation, and promote nutrient transport and metabolic waste removal in bone tissue [45, 46]. In TCM, the spleen governs transportation and transformation [17]. Spleen transforms food into nutrients, which are the sources of blood. Nutrient absorption also depends on the transporter function of the spleen [17]. Lipid metabolism disorder is often related to insulin resistance and dysregulation of gut microbiota, so it hinders nutrient absorption and affects spleen function [47, 48]. Other reported spleen‐strengthening drugs have shown the significant function of regulating lipid metabolism homeostasis [49, 50]. The kidney is also responsible for the functions and activities of bones and marrow [11, 12], and strengthening kidney alleviate osteoporosis [15, 16]. Lipid metabolism disorder and lipid peroxidation are the main causes of kidney dysfunction [51, 52]. The Chinese medicine for tonifying the kidney has shown the functions of anti‐oxidation and lipid metabolism regulation [53]. Therefore, in the treatment of osteoporosis, DG, FL, and NX may affect blood vessel function, oxidative stress, and nutrient absorption by regulating lipid metabolism, and finally play the role of invigorating blood, strengthening the spleen, and tonifying thekidneys.

Distinct lipid metabolic pathways lead to osteoporosis in different biological processes [54, 55, 56]. Phospholipid metabolism is an important part of lipid metabolism and can be divided into glycerophospholipid and sphingolipid metabolisms. In our serum metabolomics results, seven common differential metabolites between DG, FL, and NX groups were identified. They were 2,6‐dihydroxybenzoic acid, PI 39:5; PI (17:0/22:5), PI 40:7; PI (18:1/22:6), yucalexin A19, trilobinone, PI 39:4; PI (19:0/20:4), PI 36:1; PI (18: 0/18:1), most of which are associated with glycerophospholipid and sphingolipid metabolisms. In terms of bone metabolism, glycerophospholipid metabolism regulates the differentiation of bone marrow stromal cells (BMSC) into osteoblasts and maintains bone homeostasis [57]. Sphingolipid metabolism is not only involved in skeletal development, mineralization, and bone mass regulation, but also regulates the maturation and migration of osteoclast precursors [58, 59]. Therefore, DG, FL, and NX may affect the differentiation of osteoblasts and osteoclasts by regulating lipid metabolism, ultimately maintaining bone homeostasis and alleviating PMOP.

In this study, compared with the sham group, rats in the model group showed a significant increase in body weight and 24‐h urine output, while there was a decrease in rectal temperature, which were mutually consistent with the characteristics of kidney deficiency syndrome described in the Nei Jing [60]. According to the TCM theory, kidney deficiency is one of the most common etiologies of OP [61, 62]. In this study, gross pathology evaluation indicated that these three drugs relieved the symptoms of kidney deficiency. Our micro‐CT and mechanical experiments demonstrated that all three drugs could delay bone loss, improve bone mechanical properties, and enhance bone resistance to external impact in model rats. The above results suggest that the three drugs for osteoporosis may have a relieving effect on the symptoms of kidney deficiency.

Our data showed that FL and NX were closely related to amino acid metabolism. Tryptophan is an essential amino acid in the human body. It has been shown that upregulation of tryptophan metabolism leads to increased resorption by osteoclasts, which results in substantial bone loss, as well as affecting osteogenic differentiation of BMSC [63, 64, 65]. Ling et al. [65] found that serum leucine and valine levels were correlated with intestinal flora and negatively correlated with the prevalence of osteoporosis. In addition, Amy Jennings et al. showed that leucine intake was closely related to the increase of BMD of the spine and femur [66]. In this study, we found significantly decreased levels of valine and leucine in the OVX group compared with the normal group through serum metabolomics analysis, which was similar to the results of previous studies. Therefore, our findings suggest that the anti‐osteoporosis efficacy of FL and NX may be partially on the basis of their impact on intestinal flora and osteogenic differentiation of BMSC.

In our results, NX treatment also affected purine metabolism in OVX rats. Purines, which constitute the genetic material and energy unit (ATP) of living organisms, are an important class of metabolites. Generally, as the age increases, the ability to replicate genetic material, the efficiency of genetic information transfer, and ATP synthesis decline. Slower turnover of genetic material is detrimental to bone remodelling, and decreased synthesis of energy material reduces bone strength [67]. Disturbances in purine metabolism have been observed in the development of osteoporosis [68]. These evidences fully confirm that purine metabolism plays an important role in bone remodelling. Therefore, NX may also maintain osteoblastic homeostasis through purine metabolism, thereby preventing bone loss.

However, in the present study, we detected 47 metabolites between the model and sham group. These metabolites were slightly different from other previous studies. In the study of Li et al. [36], 30 metabolic differences were detected, and the changes of metabolites were mainly related to fatty acids, amino acids, choline, arachidonic acid, and taurine. This is somewhat different from our results. In our metabolic profile analysis, the samples were tightly clustered in the scatterplot of PCA scores of the QC samples, and the Pearson's correlation analysis plots of the QC samples showed good reproducibility, suggesting that the acquisition methodology and our data were stable and reliable (Figure S2). Therefore, the difference between the results of our study and those of previous studies may be related to the variant experimental conditions. Firstly, SD rats came from different companies. Li et al. bought SD rats from Changsheng Company, while we bought from Slack Company; secondly, the modelling time is different. Li et al. constructed tha OVX rat model for 14 weeks, while we built the rat model for 12 weeks. Finally, there is a difference in the type of instrument used for UPLC/Q‐TOF‐MS. Li et al. used Xevo G2‐XS Q‐TOF, while we used Triple TOF 6600. These variant experimental conditions may be responsible for differences in findings between our and the previous study. In addition, our research on the “different treatments for same disease” theory only at the level of differential metabolites in rat PMOP models. Thus, we will expand the sample size and collect more complete clinical samples for further verification in our future study.

## 5 Conclusion

The results of this study showed that DG, FL, and NX had significant efficacy in ameliorating bone loss in PMOP rat models, by regulating lipid metabolism, most of which were related to glycerophospholipids and sphingolipids. Meanwhile, FL also affected the metabolism of amino acids, and NX also influenced the metabolisms of amino acids and purine. In summary, our findings provide the biological evidence for the TCM principle of “different treatments for same disease.”

## Author Contributions

**Jingyuan Wen:** data curation (equal), methodology (equal), software (equal), validation (equal), visualization (equal), writing – original draft (equal). **Xuefeng Li:** software (equal), supervision (equal), validation (equal), visualization (equal). **Zhen Wu:** methodology (equal), software (equal), supervision (equal), validation (equal), visualization (equal). **Liu Jiangyuan:** validation (equal), writing – review and editing (equal). **Guanyin Wang:** data curation (equal), formal analysis (equal), methodology (equal), software (equal), validation (equal), visualization (equal). **Xu Wang:** software (equal), validation (equal), visualization (equal). **Zhengsheng Bao:** methodology (equal), software (equal), validation (equal), visualization (equal). **Yang Yu:** validation (equal), visualization (equal). **Pinger Wang:** software (equal), validation (equal), visualization (equal). **Zhenyu Shi:** formal analysis (equal), methodology (equal), software (equal). **Bing Xu:** software (equal), validation (equal). **Yunhuo Cai:** supervision (equal), visualization (equal). **Hongting Jin:** resources (equal), software (equal), supervision (equal), validation (equal), visualization (equal), writing – review and editing (equal). **Jiali Chen:** supervision (equal), validation (equal), visualization (equal), writing – review and editing (equal).

## Conflicts of Interest

The authors declare no conflicts of interest.

## Supporting information


**Figure S1** H&E staining results of liver and kidney. (A) Histopathological changes in liver tissue; (B) histopathological changes in renal tissue.
**Figure S2** Pearson’s correlation coefficient analysis of the abundance of QC samples after quality control.

## Data Availability

The data that support the findings of this study are available from the corresponding author upon reasonable request.
