# Supplementary figures and images for: The Mechanism Exploration of Traditional Chinese Medicine's “Different Treatments for Same Disease” Concept in Osteoporosis Therapy: A Serum Metabolomics Study
Source: J Cell Mol Med. 2025 Jul 2;29(13):e70662. doi: 10.1111/jcmm.70662 (PMC12217658; doi:10.1111/jcmm.70662)

**Supplementary Figure 1**

**
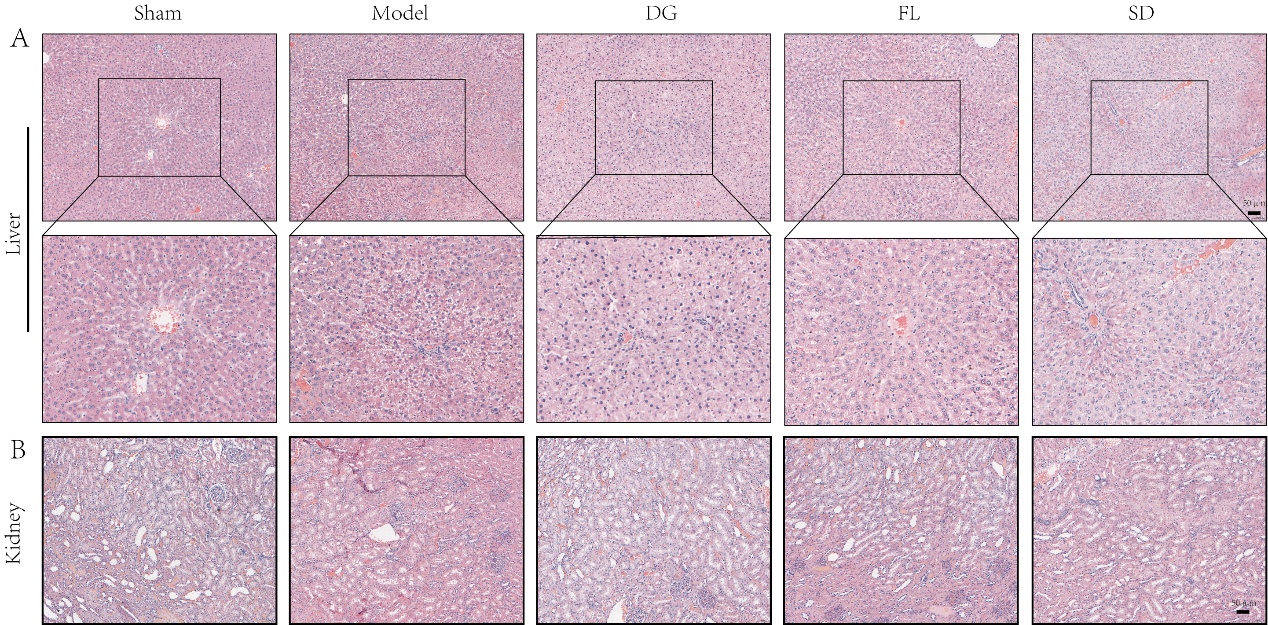
**

**Supplementary Figure 2**

**
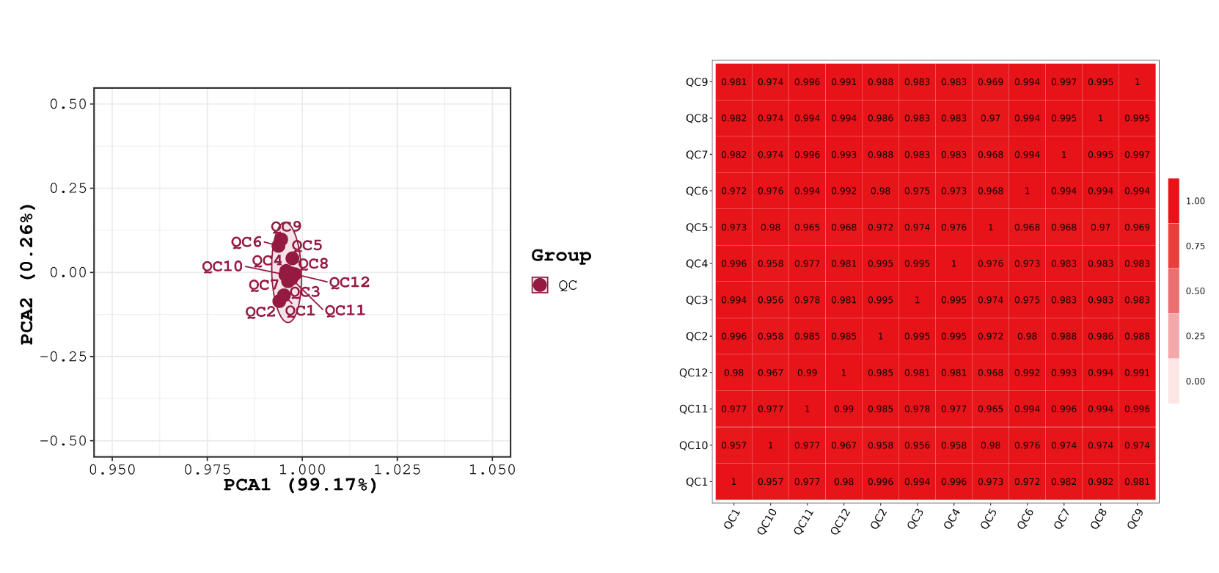
**

Supplement: Supplementary file 1 — Figure S1 H&E staining results of liver and kidney. (A) Histopathological changes in liver tissue; (B) histopathological changes in renal tissue. Figure S2 Pearson’s correlation coefficient analysis of the abundance of QC samples after quality control. [file JCMM-29-e70662-s001.docx]
